# Two strategies to improve the supply of PKS extender units for ansamitocin P-3 biosynthesis by CRISPR–Cas9

**DOI:** 10.1186/s40643-022-00583-7

**Published:** 2022-08-29

**Authors:** Siyu Guo, Xueyuan Sun, Ruihua Li, Tianyao Zhang, Fengxian Hu, Feng Liu, Qiang Hua

**Affiliations:** 1 State Key Laboratory of Bioreactor Engineering, East China University of Science and Technology, 130 Meilong Road, Shanghai, 200237 China; 2 Shanghai Collaborative Innovation Center for Biomanufacturing Technology, 130 Meilong Road, Shanghai, 200237 China

**Keywords:** *Actinosynnema pretiosum*, CRISPR–Cas9, Bidirectional promoters, Ansamitocin P-3 (AP-3), Extender unit

## Abstract

Ansamitocin P-3 (AP-3) produced by *Actinosynnema pretiosum* is a potent antitumor agent. However, lack of efficient genome editing tools greatly hinders the AP-3 overproduction in *A. pretiosum*. To solve this problem, a tailor-made pCRISPR–Cas9apre system was developed from pCRISPR–Cas9 for increasing the accessibility of *A. pretiosum* to genetic engineering, by optimizing *cas9* for the host codon preference and replacing pSG5 with pIJ101 replicon. Using pCRISPR–Cas9apre, five large-size gene clusters for putative competition pathway were individually deleted with homology-directed repair (HDR) and their effects on AP-3 yield were investigated. Especially, inactivation of T1PKS-15 increased AP-3 production by 27%, which was most likely due to the improved intracellular triacylglycerol (TAG) pool for essential precursor supply of AP-3 biosynthesis. To enhance a “glycolate” extender unit, two combined bidirectional promoters (BDPs) *ermEp-kasOp* and *j23119p-kasOp* were knocked into *asm12-asm13* spacer in the center region of gene cluster, respectively, by pCRISPR–Cas9apre. It is shown that in the two engineered strains BDP-ek and BDP-jk, the gene transcription levels of *asm13*-*17* were significantly upregulated to improve the methoxymalonyl-acyl carrier protein (MM-ACP) biosynthetic pathway and part of the post-PKS pathway. The AP-3 yields of BDP-ek and BDP-jk were finally increased by 30% and 50% compared to the parent strain L40. Both CRISPR–Cas9-mediated engineering strategies employed in this study contributed to the availability of AP-3 PKS extender units and paved the way for further metabolic engineering of ansamitocin overproduction.

**Graphical Abstract:**

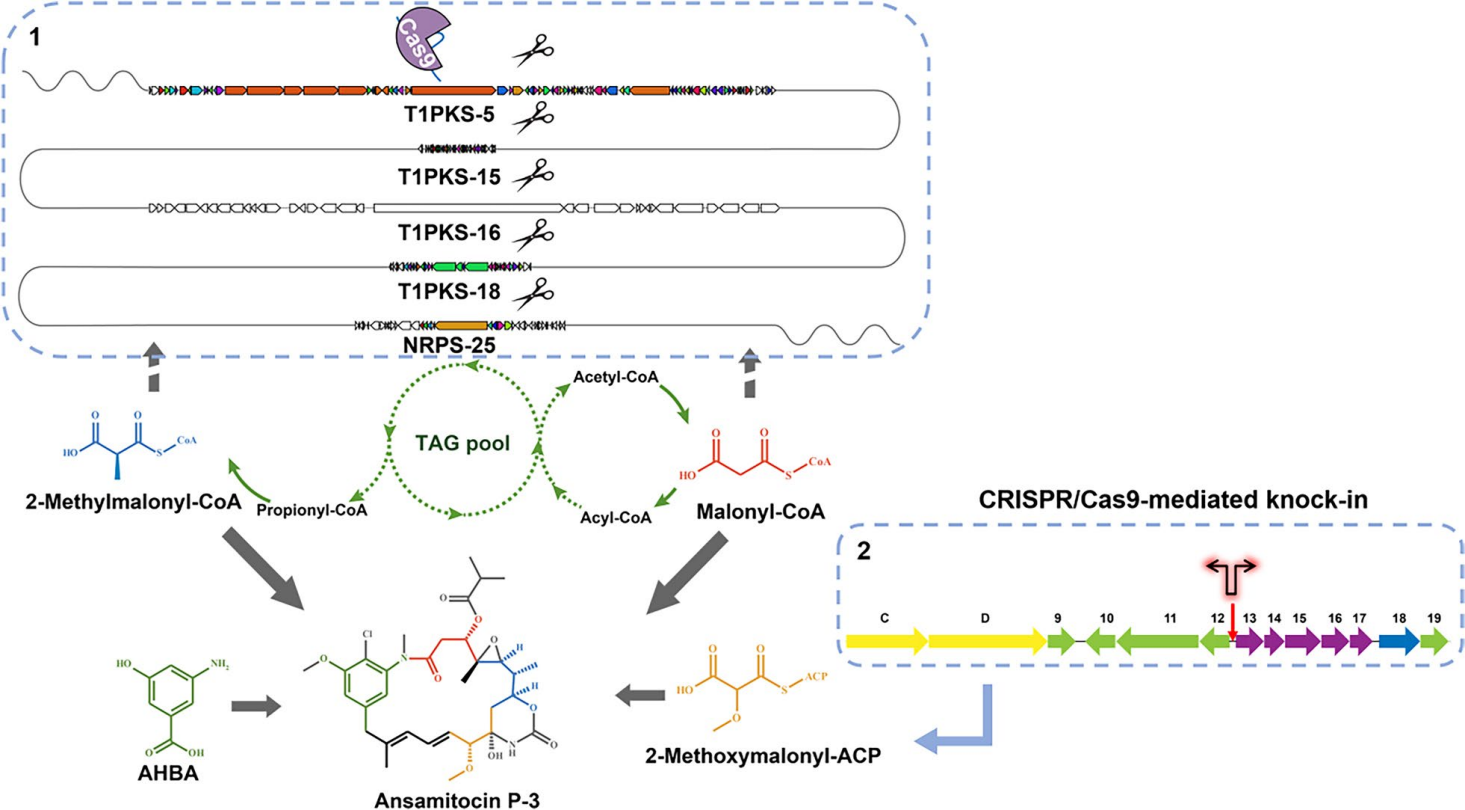

**Supplementary Information:**

The online version contains supplementary material available at 10.1186/s40643-022-00583-7.

## Introduction

*Actinobacteria*, including numerous genera, are capable of producing a wide variety of secondary metabolites with diverse bioactivities for multiple pharmaceutical applications (Bérdy 2005, 2012; Hwang et al. 2014). These valuable producers are widely utilized in academic research and industrial production (Newman and Cragg 2016; Genilloud 2017). For excellent performance in production, rational modification methods have been developed to provide a basis for metabolically engineering strategies (Wriessnegger and Pichler 2013; Kim et al. 2016). For instance, multiplex site-specific genome engineering (MSGE), based on the “one integrase-multiple *attB* sites” concept, provides a method to replace redundant gene clusters by multi-copy biosynthetic gene clusters of the target product (Li et al. 2017). Generally speaking, genetic manipulation of *Actinobacteria* heavily relies on accessible, simple, and efficient genetic tools. In the last decade, CRISPR/Cas technologies have emerged as a powerful tool for genome editing in mammalian cells, plants, and microorganisms (Alberti and Corre 2019). With high efficiency, single-gene disruptions and multiplex chromosomal deletions can be achieved in model and non-model *Streptomyces* species (Zeng et al. 2015; Huang et al. 2015; Cobb et al. 2015; Jia et al. 2017; Tong et al. 2015). However, it still requires the systematic optimization of each component in CRISPR–Cas9 system for efficiently engineering some non-model microorganisms, such as *Actinosynnema* spp.

*Actinosynnema* spp. with a truly high guanine-cytosine (GC) content (73.9%) is well known for producing ansamitocin, as well as a variety of secondary metabolites such as actinosynneptide and dnacin (Martin et al. 2014; Wang et al. 2019a; Zhong et al. 2019; Kashyap et al. 2019; Hu et al. 2020). *Actinosynnema* genome editing relies on the homologous recombination either via single or double-crossover events, hence its genetic modification is time- and labor-intensive (Yu et al. 2002; Fan et al. 2016b; Ning et al. 2017; Wang et al. 2020b). Therefore, developing a tailor-made gene editing tool for *Actinosynnema* spp. is urgent for rapid strain modification by editing multiple genes or large gene fragments in *Actinosynnema* genomes.

**Fig. 1:**
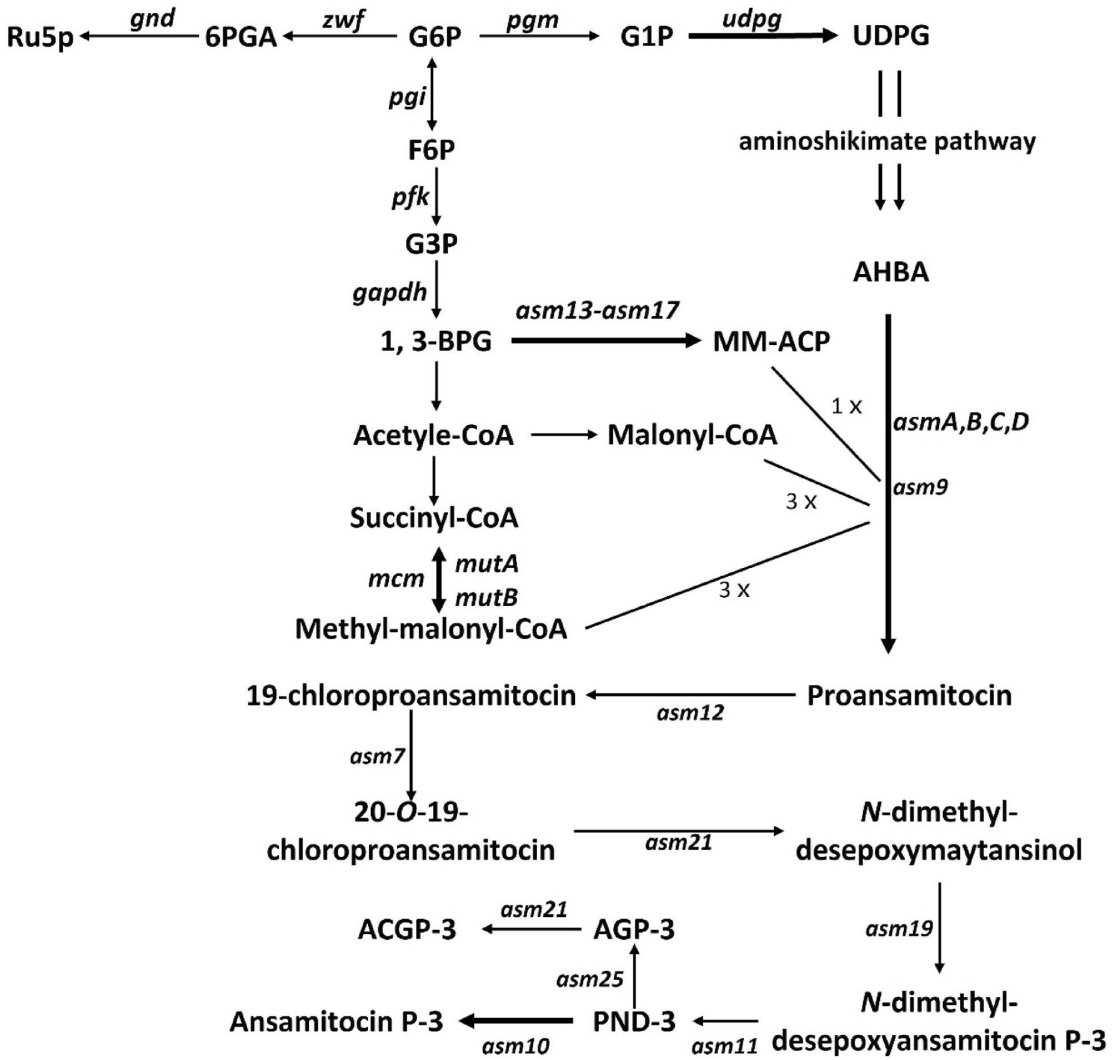
The general pathway map of AP-3 biosynthesis. *Ru5p* ribose 5-phosphate; *6PGA* 6-phosphogluconic acid; *G6P* glucose-6-phosphate; *G1P* glucose 1-phosphate; *UDPG* uridine diphosphate glucose; *F6P* fructose-6-phosphate; *G3P* glyceraldehyde 3-phosphate; *1,3-BPG* 1,3-bisphosphoglycerate; *MM-ACP* methoxymalonyl-ACP; *AHBA* 3-amino-5-hydroxybenzoic acid; *PND-3* *N*-demethylansamitocin P-3; *ACGP-3* 4″-*O*-carbamoylansamitocinoside P-3; *AGP-3* ansamitocinoside P-3

The ansamitocin biosynthesis gene cluster was identified, and the biosynthesis mechanism for this antitumor agent was proposed (Fig. 1) (Yu et al. 2002). As a typical type-I PKSs, there are four large ORFs (*asmA–D*) in the gene cluster, which involve in seven condensation steps for biosynthesis of proansamitocin using three malonyl-CoAs (M-CoAs), three methylmalonyl-CoAs (MM-CoAs), and one methoxymalonyl-acyl carrier protein (MM-ACP), with 3-amino-5-hydroxybenzoic acid (AHBA) as the starter unit (Kang et al. 2012). The final product, ansamitocin, is then obtained after six tailoring steps (Moss et al. 2002; Spiteller et al. 2003; Zhao et al. 2008). M-CoA, the most common extender unit (loading element of polyketide skeleton) (Chan et al. 2009), is predominantly produced by carboxylation of acetyl-CoA, but also by activation of malonate with M-CoA synthase (Staunton and Weissman 2001; Tong 2005; Milke and Marienhagen 2020). MM-CoA is mainly derived from reversible isomerization of succinyl-CoA and carboxylation of propionyl-CoA originating from cholesterol and fatty acids degradation (Reeves et al. 2006). Asm13-17 converts 1,3-bisphosphoglycerate (1,3-BPG) to MM-ACP, a substrate for the formation of an unusual glycolate unit in AP-3 biosynthesis (Wenzel et al. 2006). It indicates that the supply of unusual glycolate unit may contribute significantly to the increase of AP-3 biosynthesis (Du et al. 2017; Du and Zhong 2018).

The biosynthesis of secondary metabolites can be hindered by a particular extender unit shortage (Ding et al. 2015). In the past, there has been considerable interest in increasing the supply of extender units to improve polyketide yields (Reeves et al. 2006; Zabala et al. 2013). Two strategies have been typically used to increase the intracellular CoA-ester levels and concentration of polyketides. One is to overexpress the biosynthetic genes for specific extender units. For example, this approach effectively increased the production of actinorhodin, FK506, epothilone B, and AP-3 by 6-, 2-, 2.5-, and 3-fold, respectively (Stassi et al. 1998; Ryu et al. 2006; Mo et al. 2009; Zabala et al. 2013; Zhao et al. 2017). The other is to delete the putative competing gene clusters for releasing more polyketide extender units. Different studies demonstrated that it is possible to elevate the yields of polyketides by replacing genes for the biosynthesis of starter unit (Xiong et al. 2006) or by interrupting the conflicting secondary metabolite pathways (Lu et al. 2016).

Our previous studies have shown that enhancing the glucose-1-phosphate and UDP-glucose pools, as well as redirecting the flux from pentose phosphate (PP) pathway to AHBA biosynthesis, could partly increase the AP-3 production (Fan et al. 2016a, 2016b). We hypothesize that alternative gene targets for metabolic engineering modification favoring AP-3 overproduction might be involved in the secondary metabolism of *A. pretiosum*. Since then, the genome of ansamitocin producer *A. pretiosum* ATCC 31565 was fully sequenced, shedding lights on astounding productivity of ansamitocin P-3. Herein, we described the development of a CRISPR/Cas9-based genome editing tool in *A*. *pretiosum*. With this powerful tool, tandem deletion of competing gene clusters and site-specific insertion of bidirectional promoters (BDPs) successfully led to the overproduction of ansamitocin P-3 by promoting the CoA-esters accumulation and methoxymalonyl-ACP biosynthesis.

## Results

### Enabling efficient function of Cas9-sgRNA complex in *A. pretiosum*

To harness the CRISPR/Cas system for genome editing in *Actinosynnema* spp., the pCRISPR–Cas9apre was designed. The temperature-sensitive plasmid pCRISPR–Cas9 (GenBank ID: KR011749), which drives Cas9 protein expression in an inducible form, was chosen as the backbone. Given the codon usage and promoter efficiency of *Actinosynnema* spp., a codon-optimized *cas9* was under controlled by an inducible promoter tipAp*.

**Fig. 2:**
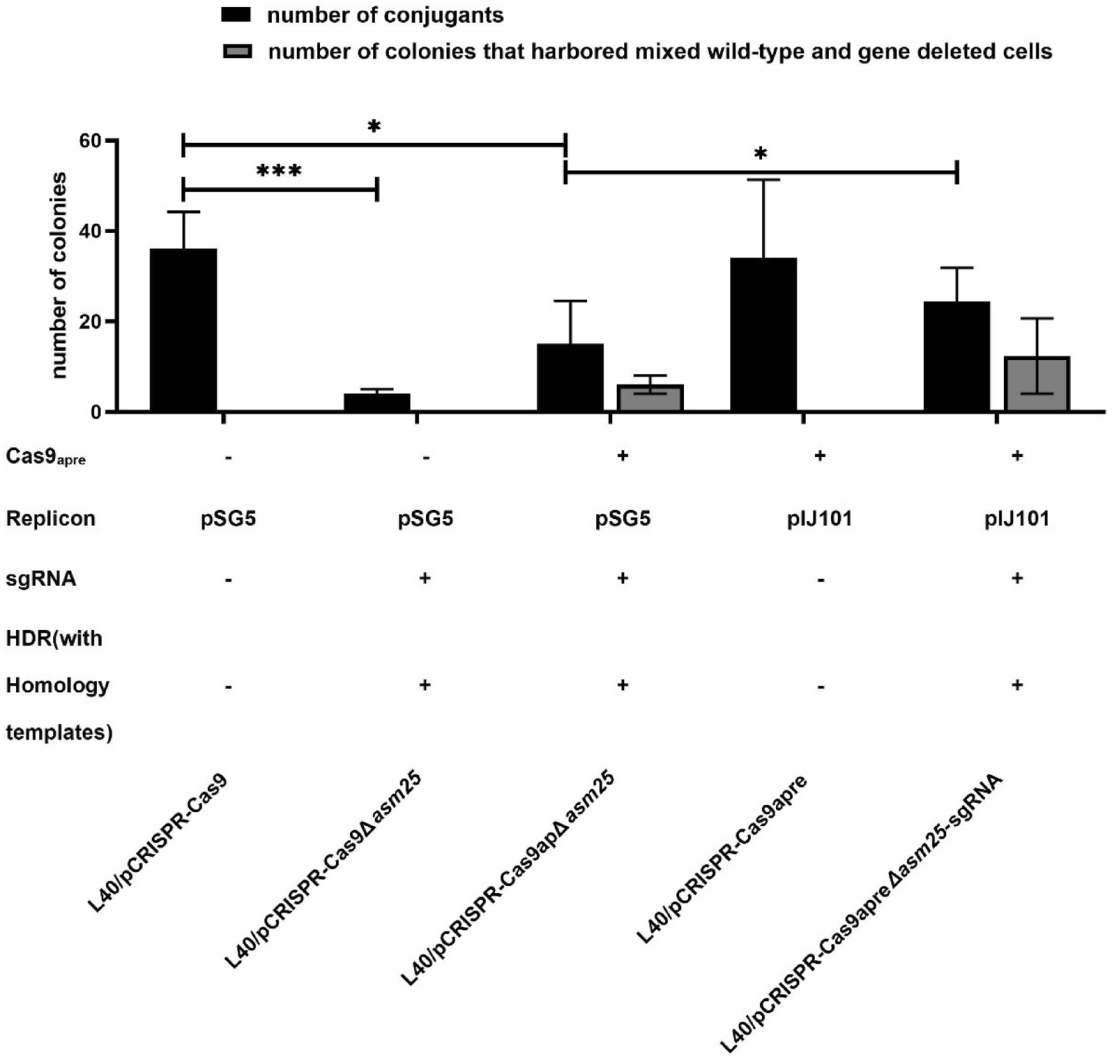
Conjugation transfer efficiency of optimized CRISPR–Cas9 plasmids. Data are means ± SD (standard deviation) from three independent conjugations. The total colonies were counted directly. Significant differences were analyzed by two-way ANOVA, and *p < 0.05, ***p < 0.0005

To examine the activity of Cas9-sgRNA in vivo, we chose to inactivate a post-PKS modification gene *asm25* that encodes an *N*-glycosyltransferase responsible for the *N*-glycosylation of *N*-demethylansamitocins (PNDs). The *N*-glycosyltransferase also competes for the *N*-demethyl-AP-3 (PND-3) with AP-3 biosynthesis (Additional file 1: Fig. S1) (Zhao et al. 2008; Ning et al. 2017). The sgRNA was identified with ApE software (http://ape-a-plasmid-editor.wikispaces.com) by analyzing the entire open reading frame of *asm25*. As negative controls, empty vector pCRISPR–Cas9 and pCRISPR–Cas9Δ*asm25* (containing the 20 nt target sequence of sgRNA and template for HDR) were, respectively, transformed into the high-yield strain L40. pCRISPR–Cas9apΔ*asm25* (containing the codon-optimized *cas9*, the target sgRNA and template for HDR) was transformed into the same parent strain for gene inactivation. Unfortunately, few conjugants were obtained whether or not the *cas9* sequence was optimized. By inducing Cas9 expression, nearly one-third of the colonies remained when templates of homologous recombination were present. Recent study shows that random gene recombination of PKS gene occurs as adopting temperature-sensitive replication-dependent vectors (with pSG5 replicon) in PKS gene knock-out experiments (Wlodek et al. 2017). Additionally, it was assumed that the pSG5 replicon might limit the accuracy of gene editing (Mo et al. 2019). Hence, we employed the structurally stable but genetically unstable replicon of pIJ101 to replace the temperature-sensitive replicon, and obtained the pCRISPR–Cas9apre. The number of pCRISPR–Cas9apreΔ*asm25-*sgRNA (containing the same sgRNA and template for HDR) (Additional file 1: Fig. S2) conjugants increased by 60% compared to that of pCRISPR–Cas9apΔ*asm25* (Fig. 2)*.* More conjugants were collected with the presence of HDR, suggesting that the incomplete Non-Homologous End Joining (NHEJ) pathway in *Actinosynnema* spp. failed to repair the DNA double strand breaks (DSBs) (Additional file 1: Fig. S3).

**Fig. 3:**
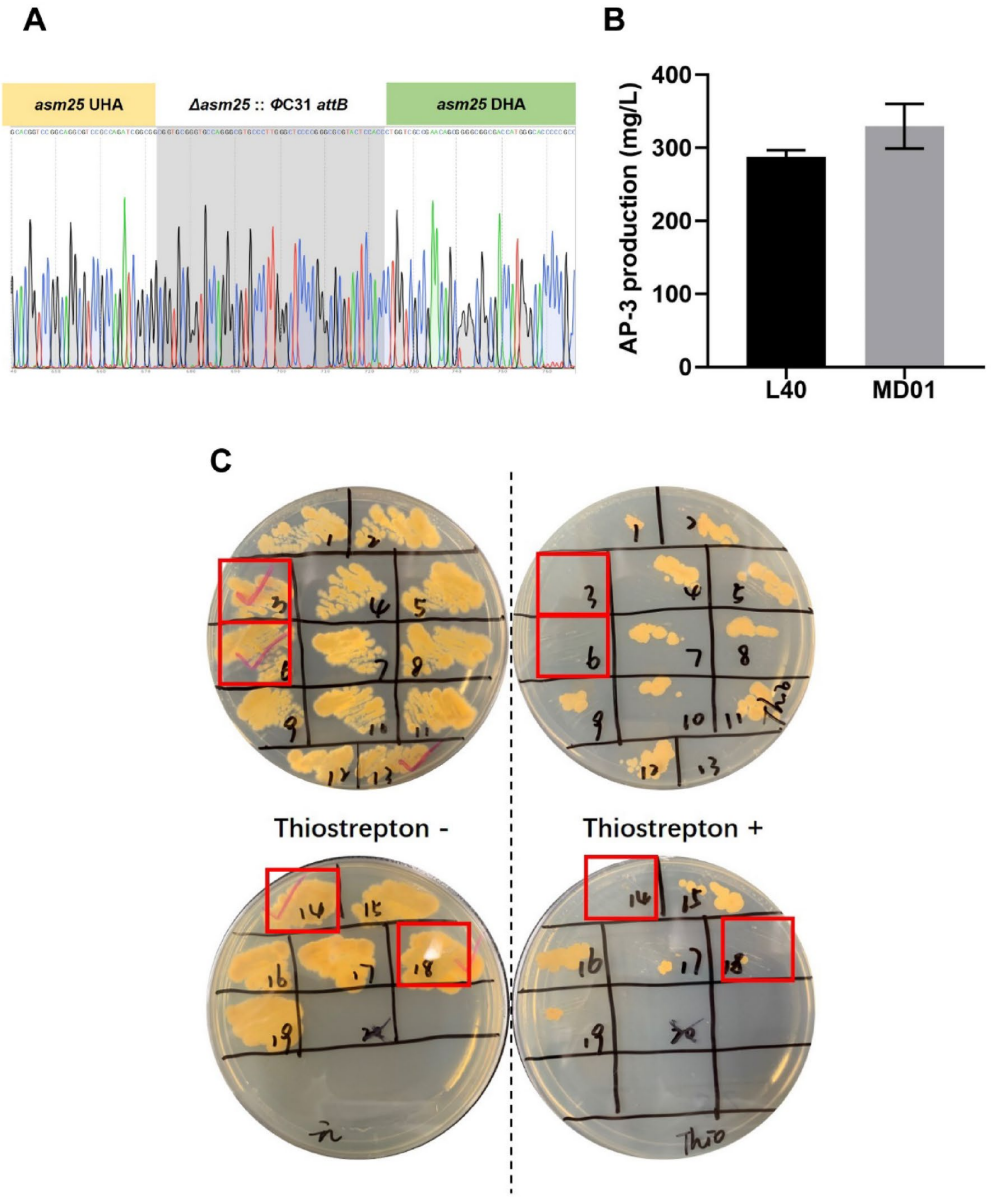
CRISPR–Cas9 system mediated *asm25* gene inactivation in *A. pretiosum*. **A** Sanger sequencing chromatograms for mutant MD01, the *Φ*C31 *attB* replaced *asm25* gene sequence showing in grey. **B** Comparison of AP-3 production of mutant MD01 and parent strain L40. MD01, mutant with *asm25* deletion. A roughly 14.5% increase in AP-3 production for MD01 compared to that of strain L40. **C** Marker-free mutant screening. Colonies were inoculated into YMG plate with thiostrepton and without antibiotics, respectively, to verify the curing of plasmid

**Fig. 4:**
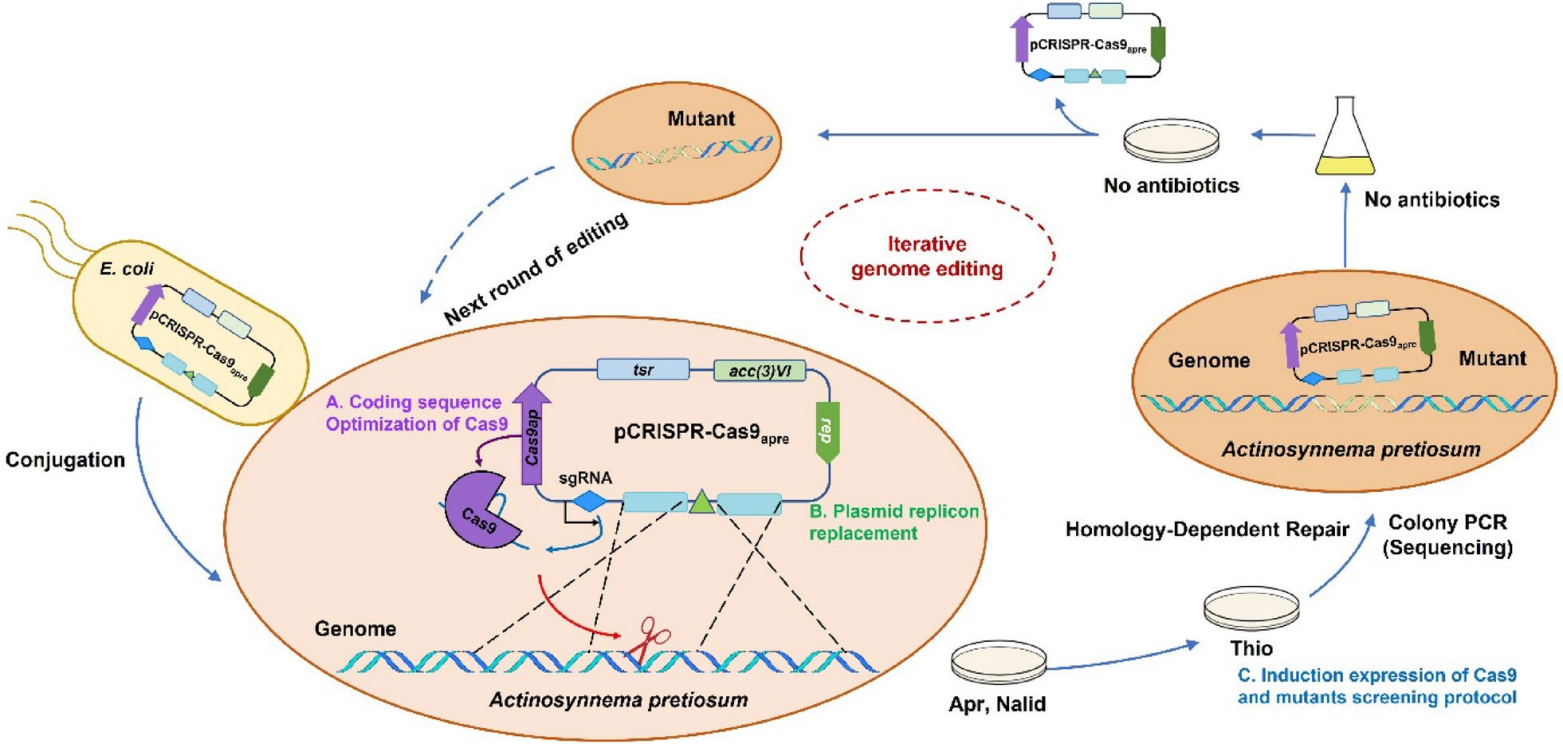
Scheme of iterative genome editing in *A. pretiosum*

Subsequently, the positive colonies were confirmed by sequencing (Fig. 3A). A *Φ*C31 *attB* site was inserted in *asm25* locus. The AP-3 production was increased to 329.2 ± 30.5 mg/L in *asm25*-mutant MD01 (Fig. 3B) owing to the interruption of competition utilization of PND-3. Plasmids in mutants were lost by three rounds (24 h per round) of liquid subculturing nonselectively (Fig. 3C). Thus, an iterative protocol was developed for multiple gene editing by pCRISPR–Cas9apre system (Fig. 4). Editing efficiency of pCRISPR–Cas9apre varied from 30 to 100% according to the N20 sequence sgRNAs (Additional file 1: Table S1).

### Genomic insights into *A. pretiosum* ATCC 31565

**Fig. 5:**
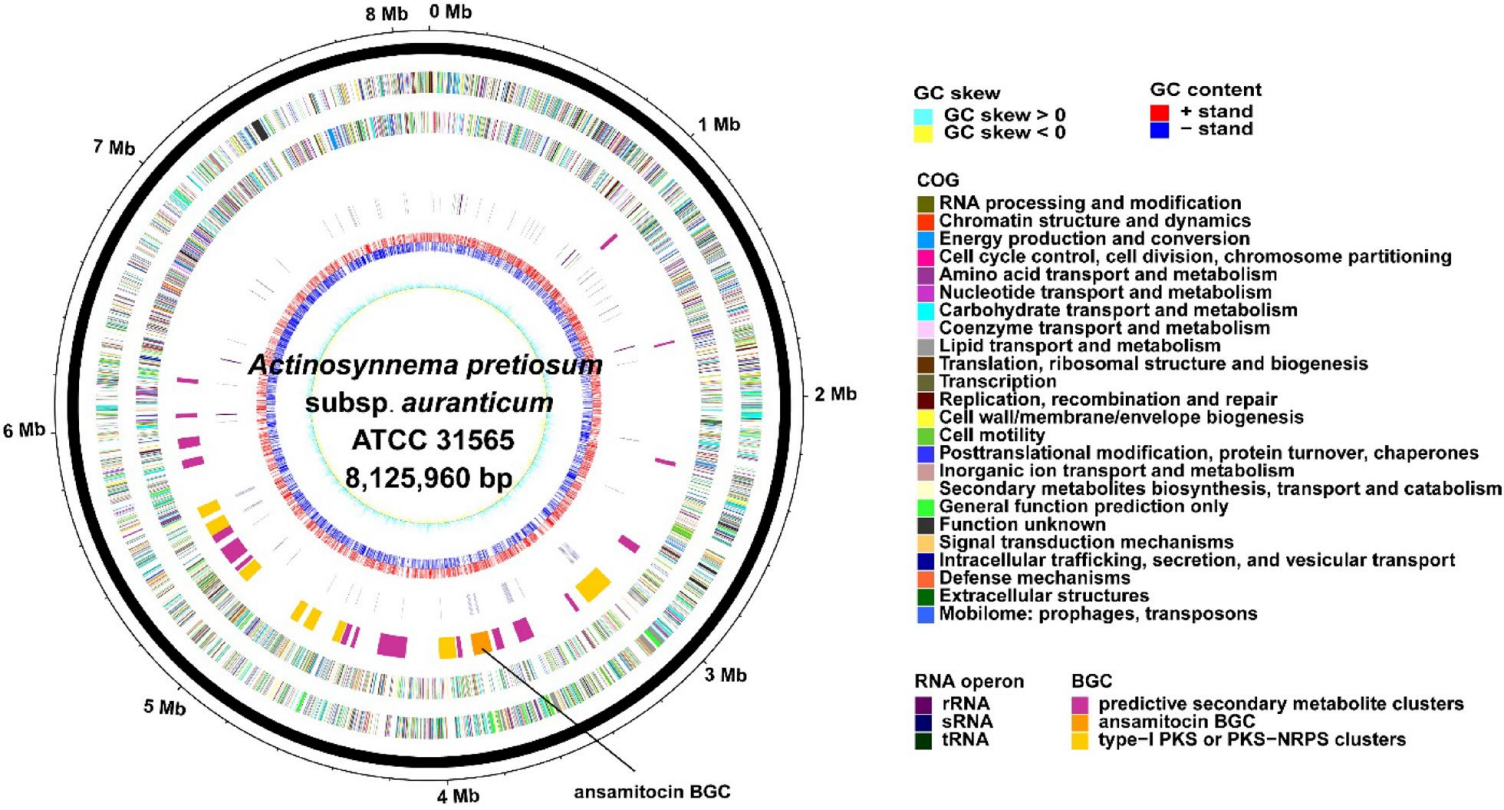
Complete genome of *A. pretiosum* subsp. *auranticum* ATCC 31565. The seven circles (from outside in) represent the genome region of ATCC 31565, CDSs on the forward strand, CDSs on the reverse strand, locations of predictive secondary metabolite clusters, distribution of rRNA, sRNA and tRNA operon, GC content and GC skew of CDSs

A circular chromosome size of 8,125,960 bp in ATCC 31565 contains 7312 CDS with an average CDS length of approximately 986 bp and a coding density of 88.7%. The G + C content of ATCC 31565 is 73.9% (Fig. 5). For mining comprehensive genetic information, two analyses were performed. The first was to construct central carbon metabolic network based on gene function prediction. ATCC 31565 has a complete primary metabolic pathway (glycolysis, tricarboxylic acid cycle, and pentose phosphate pathway). The number of relevant functional genes and their copies in the primary metabolic pathways associated with building precursors of ansamitocin are depicted in Additional file 1: Fig. S4. Compared with the model strain (such as streptomycetes), ATCC 31565 exhibits a relatively weak primary metabolism activity due to the low percentage of primary metabolism genes in its genome. Given that primary metabolism provides the precursors for secondary metabolism, improving the primary metabolism may facilitate synthesis of ansamitocin in ATCC 31565. The other analysis was to predict putative gene clusters (Additional file 1: Table S2). Twenty-seven putative gene clusters were identified by anti-SMASH, which again confirmed that Actinobacteria possesses the ability to produce a rich source of secondary metabolites. Region 9 was identified as ansamitocin biosynthetic gene cluster. Region 5 showed high homology with the reported polyene macrolide biosynthetic gene cluster *plm* (Wang et al. 2019a). Moreover, ansamitocin polyketide chain extension would require M-CoA and MM-CoA. Eight type I PKS gene clusters utilizing these acyl-CoA esters were counted based on sequence homology in the acyltransferase (AT) domain (Additional file 1: Table S3, gene cluster renamed according to the type).

### Transcription analysis and individual deletion of competing gene clusters

**Fig. 6:**
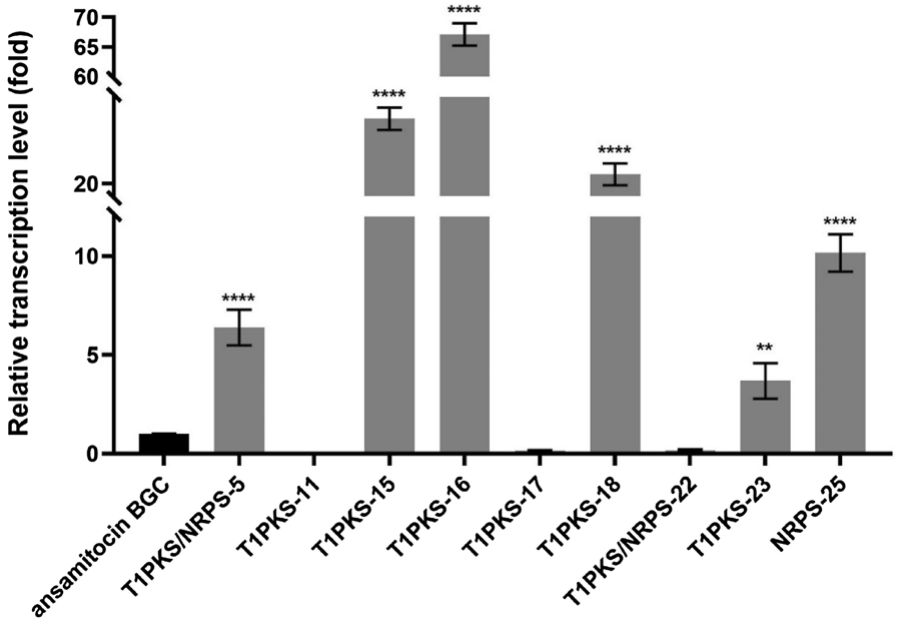
Transcription analysis of the secondary metabolic synthesis gene cluster in strain L40. Transcription analysis of the putative gene PKS clusters in strain L40. The 2^−ΔΔCt^ method was used to calculate the expression level, the average expression value of *asmC* is set to 1 as the standard. Fermentation experiments were performed three times. The values are means ± SD (standard deviation) of three independent experiments. Significant differences were analyzed by one-way ANOVA, and **p < 0.01, ****p < 0.0001

Although the mutant strains derived from atmospheric and room temperature plasma (ARTP) mutagenesis are homologous to the parental generation, there may be differences in their secondary metabolic distribution. The eight gene clusters were further determined by gene amplification in the genome of strain L40. RNA samples of L40 mycelia were taken on day 3 of cultivation and used to quantify transcript levels of the eight gene clusters by RT-qPCR. The transcription levels of T1PKS/NRPS-5, T1PKS-15, T1PKS-16, T1PKS-18, and NRPS-25 were significantly higher than that of ansamitocin biosynthetic gene cluster (Fig. 6).

**Fig. 7:**
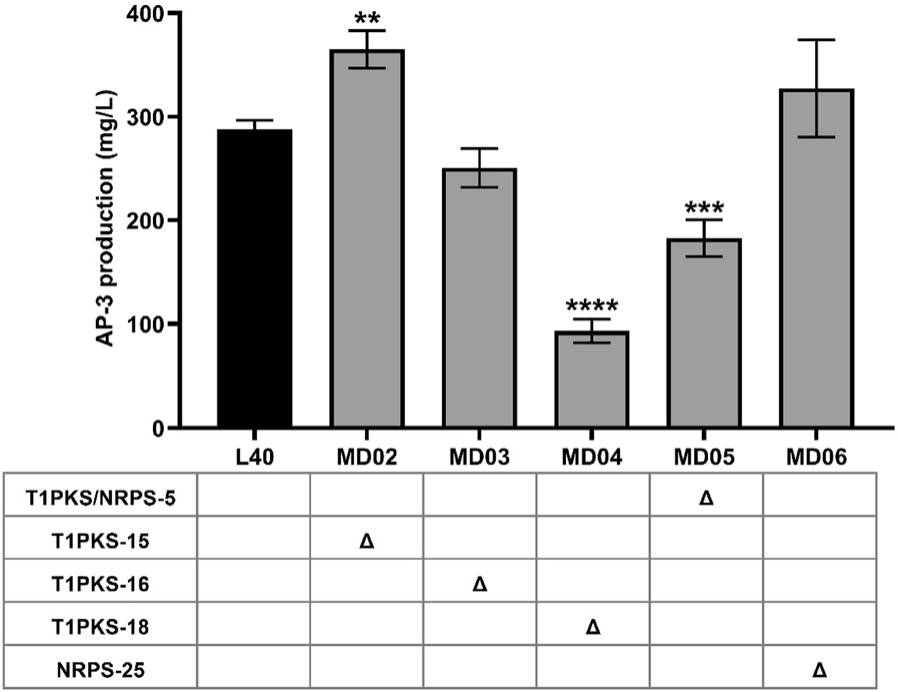
AP-3 production of mutants at the end of fermentation. L40, the parent strain. MD02, mutant with T1PKS-15 deletion. MD03, mutant with T1PKS-16 deletion. MD04, mutant with T1PKS-18 deletion. MD05, mutant with T1PKS/NRPS-5 deletion. MD06, mutant with NRPS-25 deletion, as the negative control. The fermentation experiments were performed for three times. The values are means ± SD (standard deviations) of three independent experiments. Significant differences were analyzed by one-way ANOVA, and **p < 0.01, ***p < 0.001, ****p < 0.0001

Therefore, these five gene clusters were deleted with pCRISPR–Cas9apre system individually in strain L40, generating the mutants MD02 (T1PKS-15 deletion), MD03 (T1PKS-16 deletion), MD04 (T1PKS-18 deletion), MD05 (T1PKS/NRPS-5 deletion) or MD06 (NRPS-25 deletion). To improve the genetic manipulability, artificial gene integration sites were simultaneously inserted into mutants MD02, MD03, MD04 and MD06 by template, respectively (Additional file 1: Fig. S5). Using a strategy such as MSGE, the mutant will be endowed with a multi-copy gene integration function (Li et al. 2017, 2019). AP-3 production of all mutant strains was tested on day 8 of fermentation. Surprisingly, only the mutant MD02 with the disruption of T1PKS-15 showed significant increase in AP-3 production, i.e., approximately 365 mg/L AP-3 and 27% higher than that of parent strain L40 (no significant difference was observed in dry cell weight, Fig. 7 and Additional file 1: Fig. S6). Unexpectedly, the AP-3 production of mutant strains missing the T1PKS-18 or T1PKS/NRPS-5 gene cluster reduced rapidly compared to the original strain. In particular, the loss of the T1PKS-18 gene cluster led to a dramatic two-thirds reduction in AP-3 yield of mutant MD04 (Fig. 7). The significant differences in AP-3 yields of the mutant strains implied that the synthetic precursors of AP-3 may also show drastic intracellular variations in these mutants.

### Analysis of intracellular TAG and M-CoA in PKS-disrupted mutants

**Fig. 8:**
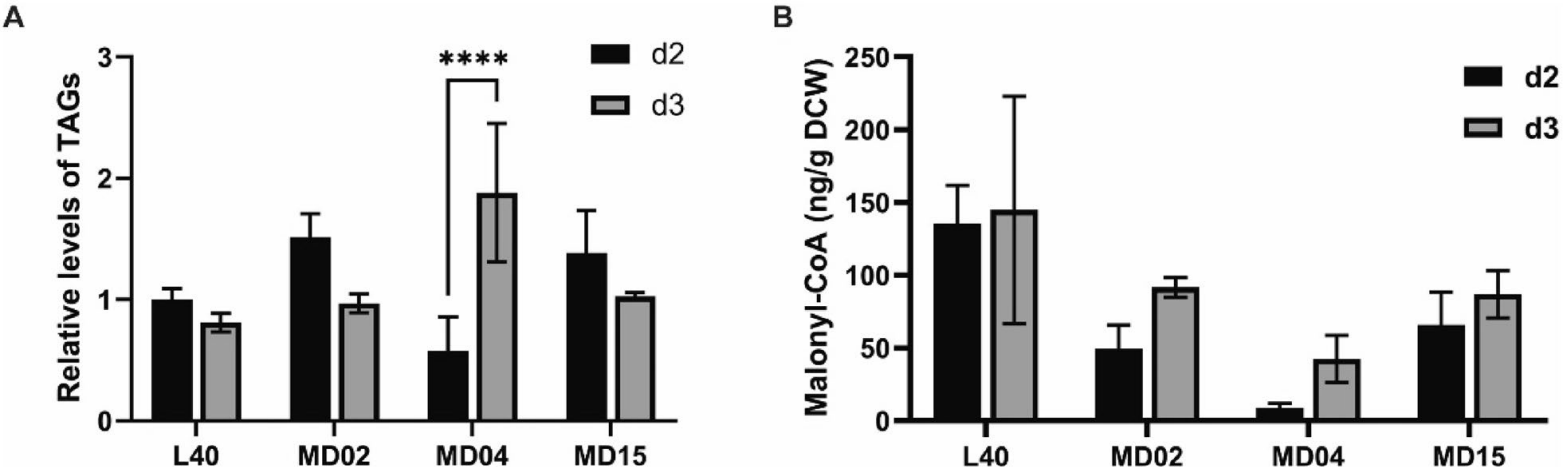
Analysis of intracellular TAG and M-CoA in PKS-disrupted mutants. **A** Relative levels of intracellular TAG pool of strain MD02, MD04, MD15 on day 2 and day 3 of fermentation. Data were normalized based on the TAG pool of strain L40 at day 2 of fermentation. The values are means ± SD of three independent experiments. Significant differences were analyzed by two-way ANOVA, and ****p < 0.0001. **B** Concentrations of the intracellular M-CoA in gene cluster-disrupted mutants. The values are means ± SD of three independent experiments. *L40* the parent strain. *MD02* mutant with T1PKS-15 deletion. *MD04* mutant with T1PKS-18 deletion. *MD15* mutant with T1PKS-15 and T1PKS/NRPS-5 deletion

Acetyl-CoA and M-CoA are important components of TAGs synthesis and accumulation in primary metabolism (Alvarez and Steinbüchel 2002; Arabolaza et al. 2008; Gomma et al. 2015). During the stationary growth phase, the carbon flux is usually channeled into polyketide biosynthesis in Actinobacteria while TAGs are generally degraded (Wang et al. 2020a). Therefore, the detection of intracellular TAG content in mutant strains may well represent a profile of its precursor supply. The intracellular TAG content of the parent strain L40 decreased by 20% from the second day to the third day of fermentation (Fig. 8A), and the degradation of TAGs may effectively promote the ansamitocin biosynthesis during this stage. The amounts of TAG accumulation on the second day and TAG degradation from day 2 to day 3 in MD02 mutant were more significant than those of the parent strain L40 and other mutants (MD04 and MD15) (Fig. 8A), which may contribute to the formation of abundant CoA-ester extender units to improve the AP-3 biosynthesis of MD02 mutant. The significantly increased intracellular M-CoA content of MD02 mutant on the third day of fermentation also partially confirmed the above hypothesis (Fig. 8B). Although MM-CoA is known to be another important PKS extender unit, we observed varying degrees of decrease in MM-CoA content in the parent strain and all mutants from day 2 to day 3 of fermentation (data not shown). This may be related to the fact that there were already sufficient MM-CoA pools in all strains during this metabolically active phase, as also mentioned elsewhere (Du et al. 2017; Du and Zhong 2018).

### Activating the MM-ACP pathway with bidirectional promoters

**Fig. 9:**
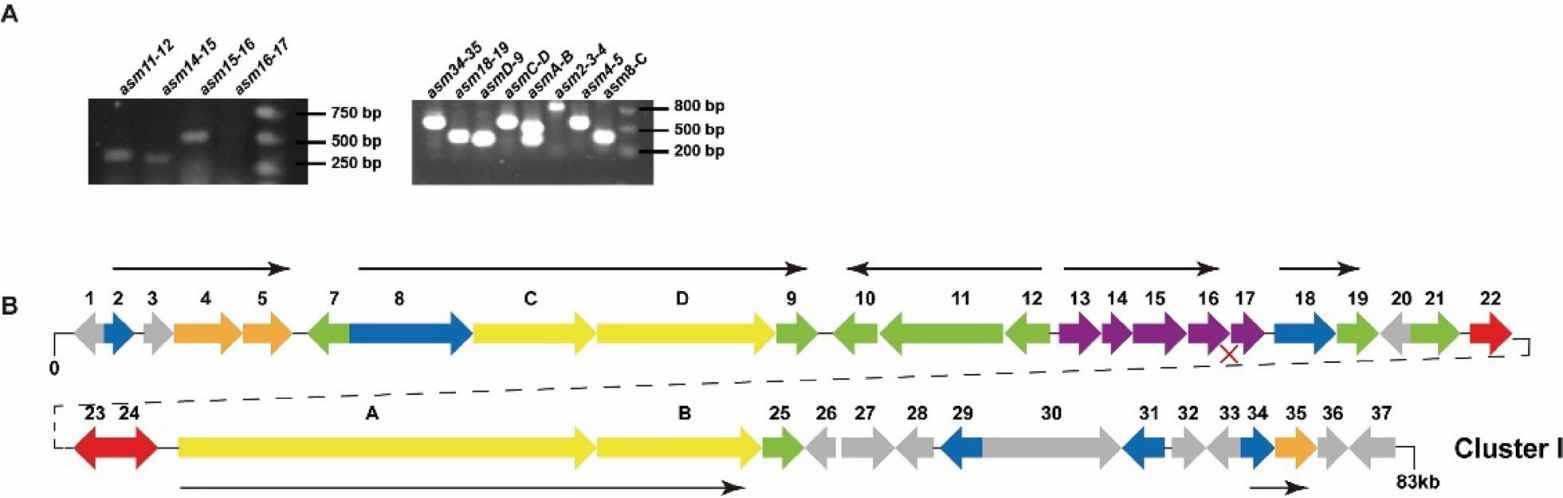
Transcript assay of AP-3 biosynthetic gene cluster (**A**) and transcription unit distribution (**B**). **A** RNA sample was isolated from day 3 culture of strain L40. RT-PCR fragments were shown. (*asm11-12*, 374 bp; *asm14-15*, 341 bp; *asm15-16*, 513 bp; *asm16-17*, 435 bp; *asm8-C*, 362 bp; *asm2-3-4*, 854 bp; *asm4-5*, 566 bp; *asmC-D*, 589 bp; *asmD-9*, 348 bp; *asmA-B*, 482 bp; *asm18-19*, 402 bp; *asm34-35*, 617 bp). **B** Horizontal black arrows show the transcription unit confirmed by RT-PCR. ‘x’, no mRNA connecting the genes. *asm11* shares 4 bp with *asm10*, *asm13* shares 4 bp with *asm14*

Genes *asm13*-*17* are located at the center of the ansamitocin biosynthesis gene cluster. We conducted gene co-transcription analysis using RT-PCR on the gene cluster. The *asm13*, *asm14*, *asm15* and *asm16* genes all belong to a transcriptional unit that does not contain *asm17*. In the opposite direction, *asm12* belongs to an operon consisting of three genes (Fig. 9). Asm12 introduces chloride onto proansamitocin as the start of post-PKS modifications. Asm11 and Asm10 catalyze the last two steps to yield AP-3 finally. Given such distribution, the spacer of *asm12* and *asm13* was selected as the target. Two combined BDPs were designed using the commonly used constitutive promoters kasOp*, ermEp* and j23119p*: *ermEp-kasOp*, *j23119p-kasOp*. Among them, kasOp* and ermEp* are stronger than any native promoters in *A. pretiosum*. Such bidirectional promoters have been used to activate target gene clusters in both *Streptomyces* and *Saccharopolyspora* species (Zhang et al. 2017; Liu et al. 2019). Two sgRNAs were designed for *asm12-asm13* spacer target sequences (Additional file 1: Fig. S7A). The plasmids were constructed according to the previous procedure (Additional file 1: Figs. S7B and C). Positive colonies were screened by PCR and verified by sequencing (Additional file 1: Fig. S8) to obtain bidirectional promoter knock-in mutants named BDP-ek and BDP-jk.

**Fig. 10:**
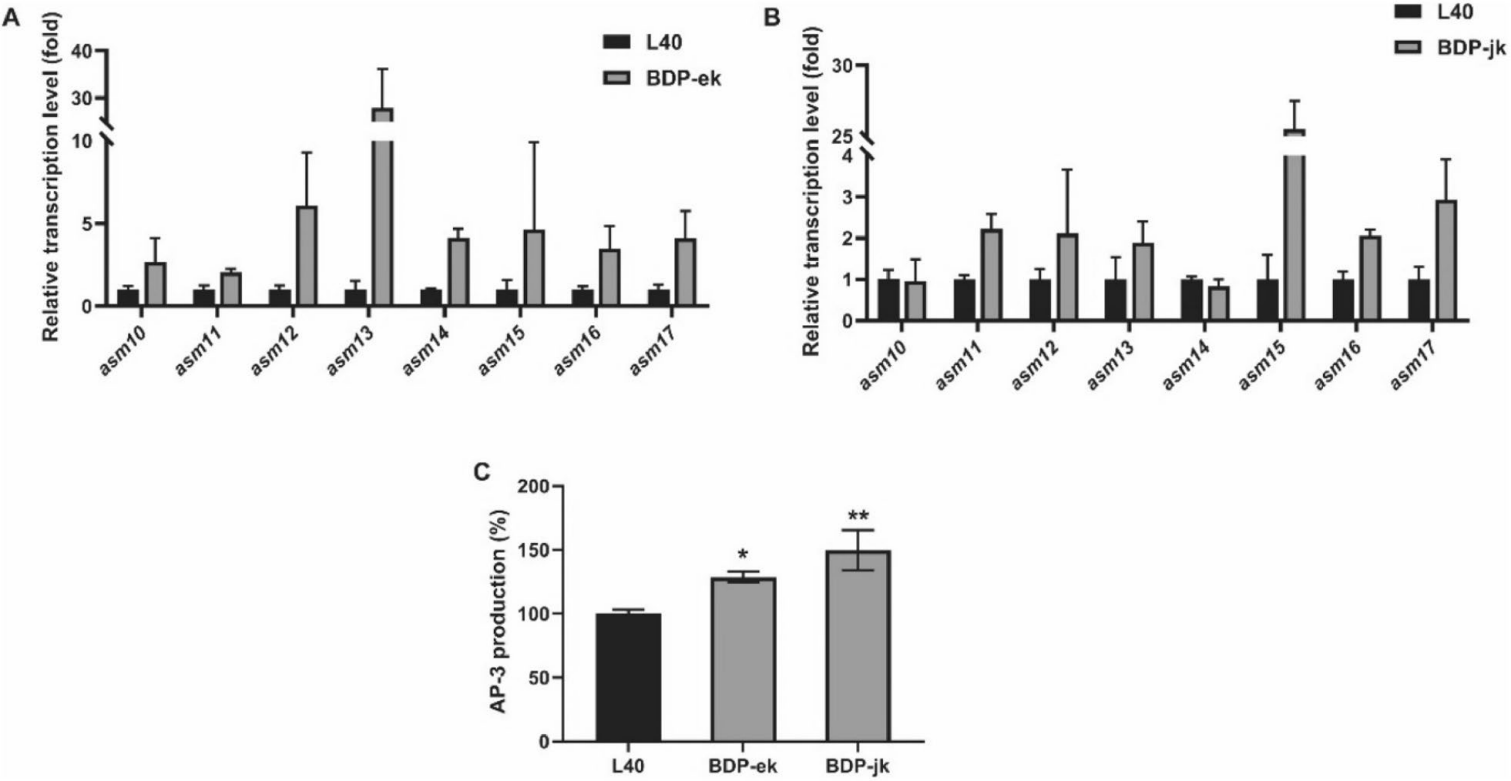
Transcription levels of AP-3 biosynthetic genes (**A**, **B**) and AP-3 production of strain L40 and bidirectional promoter knock-in mutant strains (**C**). **A**, **B** Transcription pattern of the genes (*asm10*, *11*, *12*, *13*, *14*, *15*, *16*, *17*) in bidirectional promoter knock-in mutant strains and L40 at day 3 during fermentation process. **C** Fermentation performance of control strain L40 and bidirectional promoters knock-in strains. Fermentation experiments were performed for three times. The values are means ± SD of three independent experiments. Significant differences were analyzed by one-way ANOVA, and *p < 0.05, **p < 0.005

In both recombinant strains, genes expressions were determined by RT-qPCR analysis. As expected, transcription units in both directions were significantly up-regulated. Interestingly, the transcription level of the *asm17* gene was also remarkably increased by 3- to 5-fold in both BDP-ek and BDP-jk compared with original strain L40. We also observed that the transcription elevation of the *asm14* gene was much less than its preceding gene *asm13*, indicating that the *asm14* gene may be a further modification target for MM-ACP supply. Additionally, *asm13* gene transcription was most significantly up-regulated in strain BDP-ek. While for strain BDP-jk, *asm15* behaved the most significant increase in transcriptional level (Fig. 10A and B).

The AP-3 productions of the original strain L40 and two mutant strains with the engineered bidirectional promoters were examined. An increase of 30% and 50% was obtained in mutant BDP-ek and BDP-jk, respectively. The ansamitocin yields of the mutants BDP-ek and BDP-jk increased by about 30% and 50%, respectively, largely due to the enhanced fluxes of the MM-ACP biosynthetic pathway and part of the post-PKS pathway (Fig. 10C). Although overexpression of *asm13-17* is able to enhance the MM-ACP synthesis pathway (Du et al. 2017; Du and Zhong 2018), we found that significant up-regulation of *asm15* in strain BDP-jk appeared to be more favorable for AP-3 production than significant up-regulation of *asm13* in strain BDP-ek.

## Discussion

In this study, we described an approach to develop a CRISPR/Cas9-based genome editing tool for non-model actinomycetes *A. pretiosum*. We have demonstrated its efficiency and rapidity in the development of two metabolic engineering strategies. The supply of precursors for AP-3 biosynthesis was significantly promoted by competitive cluster deletion and BDP insertion.

A prerequisite for the current use of the CRISPR–Cas9 system is the genetic tractability of the *Actinosynnema* species. Other critical issues that should be considered for CRISPR/Cas9 systems are (i) toxicity of Cas9 in the particular strain used, as reported attempts to use pCRISPomyces-2 in *Streptomyces* sp. KY 40-1 (Salem et al. 2017) and (ii) poor expression of the *cas9* gene (Alberti and Corre 2019; Tong et al. 2019). Furthermore, significantly reduced conjugation efficiency was observed when this genetic modification system was introduced into *Streptomyces* with constitutively expressed Cas9 (Zeng et al. 2015; Wang et al. 2019b; Ye et al. 2020), thus leading to the failure of genome editing. Nevertheless, controlling Cas9 expression with weak promoters at the transcriptional level or a repressive riboswitch at the translational level could be an alternative way to ameliorate the Cas9 cytotoxicity (Ye et al. 2020). Random recombination caused by pSG5-derived replicon (temperature-sensitive replication) has been observed in the engineering of both rapamycin and tylosin PKS genes (Wlodek et al. 2017). In addition, editing the *Saccharopolyspora erythraea* using the pCRISPR–Cas9 plasmid also resulted in unpredictable homologous recombination (Mo et al. 2019). In light of the above, our CRISPR–Cas9 system was modified with pCRISPR–Cas9 as a backbone harboring both Cas9 and the sgRNA driven by tipAp* and ermEp* promoter, respectively. Compared with constitutive expression of Cas9, inducible expression of Cas9 improves the transformation efficiency, which is important for subsequent positive screening and iteration (Zeng et al. 2015; Mo et al. 2019). An inducible promoter tipAp* was used to dynamically control Cas9 activity by inducer concentration (Wang et al. 2019b). The Cas9-encoding gene was codon-optimized for *Actinosynnema* spp. The pSG5-based Cas9 system may generate negative colonies with random gene recombination. The replicon can be replaced by replicon of pIJ101 as an alternative way to overcome this obstacle, probably attributed to the segregational instability of pIJ101 replicon (Mo et al. 2019). We discarded employing the counterselection marker to ensure plasmid mobility by controlling the size of the plasmid (Additional file 1: Fig. S9). An average efficiency of 77% in precise gene editing was observed in *A. pretiosum* using HDR. We further confirmed the feasibility of the iterative protocol as previously reported (Sun et al. 2009; He 2010; An et al. 2021).

Enhancing the precursors supply could be a comprehensive and promising method to promote the biosynthesis of secondary metabolites (Bilyk and Luzhetskyy 2016). Given the incomplete genomic information of AP-3 producers, previous studies were mainly focused on single or double genes in UDPG synthetic pathway and pentose phosphate (PP) pathway by the traditional gene modification method. And modulation of both pathways resulted in an increase of about 40% in AP-3 productions (Fan et al. 2016a, 2016b). With the developed genome-editing tools, we have successfully performed genome modification of AP-3 producers. One strategy is to eliminate the potential precursor competition. And deletion of T1PKS-15 resulted in a remarkable increase in AP-3 yield. However, deletion of T1PKS/NRPS-5 or T1PKS-18 failed to increase AP-3 yield. After further analyzing the transcriptions of related genes involved in UDP-glucose biosynthesis, AP-3 biosynthesis and fatty acyl-CoA accumulation in the mutants, we found that the intracellular precursor supply and the gene transcription of key pathways in deletion of T1PKS/NRPS-5 showed similar trends as those of the parent strain L40 (Fig. 8 and Additional file 1: Fig. S10). Besides, the T1PKS/NRPS-5 cluster sharing high similarity to *plm* biosynthetic gene cluster could direct to the synthesis of a polyene macrolactam, pretilactam (Wang et al. 2019a). Previous studies suggested that AP-3 shares efflux proteins with pretilactam (Wang et al. 2021), which encouraged us to hypothesize that inactivation of T1PKS/NRPS-5 may diminish AP-3 production. For the cluster T1PKS-18, quite a few similar gene clusters have been identified. In addition, unlike yield-enhancing strain MD02, in which intracellular TAG accumulated heavily at day 2, increased intracellular TAG content was observed in early stationary growth phase of mutant MD04 (Fig. 8A). Furthermore, the transcription profile of fatty acyl-CoA synthase gene of MD04 was completely opposite to that of the parent strain L40 (Additional file 1: Fig. S11), which suggested that deletion of the 50 kb gene fragment triggered the synthesis of long-chain fatty acyl-CoA. As T1PKS-18 contains a variety of functional genes responsible for polyketide formation, transcriptional regulation or transportation, screening for crucial genes need to be accomplished by individual gene deletions in subsequent studies.

Using CRISPR–Cas9-mediated promoter knock-in strategy to activate individual synthetic pathways is a common approach for enhancement of target compound yield in synthetic biology and natural product discovery (Zhang et al. 2017; Liu et al. 2019; Mo et al. 2019). In contrast to gene overexpression, bidirectional promoters (BDP) insertion allows co-expression of multiple genes and improve the flexibility of metabolic pathway optimization (Vogl et al. 2018). Here, the supply of glycolate unit is considered to be the bottleneck for AP‐3 biosynthesis (Fan et al. 2016b; Du and Zhong 2018). It has been reported that overexpression of precursor biosynthetic genes can effectively increase the yield of target metabolites under a strong promoter (Zhou et al. 2021). Motivated by the native gene distributions of *asm10-12* and *asm13-17*, we generated two recombinant strains with BDP knock-in. The transcription levels of both the MM-ACP biosynthetic genes and the tailoring genes (*asm10*, *11*, *12*) were increased, resulting in higher yields of AP-3 (Fig. 10). This strategy was firstly developed to promote gene transcriptional level in two pathway genes by a single genetic modification for overproduction of AP-3. The BDPs with different expression intensities expanded the flexibility of gene expression. Further improvement in AP-3 production with the BDP library strategy may be expected, as successfully demonstrated by optimized gene co-expression for taxadiene and β-carotene biosynthesis (Vogl et al. 2018).

On the other hand, random mutations may activate silent genes leading to splitting of precursors and carbon fluxes to other metabolisms. These changes in the mutants may introduce new limitations for AP-3 overproduction. In this study, a minimum of 27% increase in AP-3 yield was obtained based on the objective-oriented strategies. The AP-3 production obtained in mutant MD02 (365 mg/L) was higher than those recently reported, where improvement of AP-3 tolerance and enhancement of the efflux efficiency of AP-3 were mainly focused (Wu et al. 2020; Wang et al. 2021, 2020b). We additionally constructed the strain with both the knockout of gene cluster T1PKS-15 and the knock-in of BDP promoter. However, there was no further increase in yield compared with MD02 or bidirectional promoters knock-in mutants, which was probably due to the degradation of AP-3 synthesis performance of the engineered strain. Moreover, a global regulator may comprehensively control AP-3 biosynthesis during strain fermentation. Some pleiotropic transcriptional regulators have been reported to play a central role in regulation of secondary metabolism and morphological differentiation in most *Streptomyces* species (Makitrynskyy et al. 2013; Qiao et al. 2020). The limited increase of yield in this study may be caused by the restrictive regulatory pathways and this hypothesis needs to be supported by further experiments.

## Conclusion

In this study, we developed a tailored CRISPR/Cas9-based genome-editing tool allowing for scarless genome editing in *Actinosynnema*. Leveraging this versatile tool, we proposed two strategies to improve the precursor supply for AP-3 biosynthesis. For gene deletion, inactivation of competing PKS gene cluster enhanced AP-3 production by redirecting the metabolic flux of building precursors. For gene insertion, the introduction of BDPs alleviated the bottlenecks in both glycolate unit supply and tailoring steps of AP-3 biosynthesis, which therefore led to the overproduction of AP-3. The developed engineering strategies can also provide guidance to the effective construction other cell factories.

## Materials and methods

### Bacterial strains, plasmids and culture conditions

The bacterial strains and plasmids used in this study are listed in Additional file 1: Table S4. *A. pretiosum* subsp. *auranticum* L40 and its derivatives were cultivated as described previously (Li et al. 2021). In brief, strains were cultured on YMG agar plates (0.4% yeast extract, 1.0% malt extract, 0.4% glucose, 1.7% agar (w/v), pH 7.0) for the growth of aerial mycelia and TSBY liquid medium (3.0% tryptone soya broth powder, 0.5% yeast extract, and 10.3% sucrose (w/v), pH 7.2) for enrichment culture. For fermentation experiments, agar-grown mycelia were inoculated in seed medium (1.0% glucose, 0.5% yeast extract, 1.0% glycerol, 0.5% corn syrup, 1.5% soluble starch, 0.2% calcium carbonate (w/v), pH 7.0) and the fermentation medium containing 0.94% (w/v) fructose, 2.68% (w/v) glycerol, 0.3% (w/v) soluble starch, 0.7% (w/v) yeast extract, 0.1% (w/v) NH_4_Cl, 0.05% (w/v) MgSO_4_⋅H_2_O, 0.001% (w/v) FeSO_4_⋅H_2_O, 0.05% (w/v) KH_2_PO_4_, 0.5% (w/v) CaCO_3_, 2% (w/v) buckwheat flour, pH 7.4. Strains were cultured in shake flasks at 28 °C and analyzed at the end of the eighth day of fermentation.

### Genome sequencing, annotation, and analysis of *A. pretiosum *subsp*. auranticum* ATCC 31565

The genome sequencing of ATCC 31565 was sequenced using a PacBio RS II platform and Illumina HiSeq 4000 platform at the Beijing Genomics Institute (BGI, Shenzhen, China). Draft genomic unitigs, which are uncontested groups of fragments, were assembled using the Celera Assembler against a high-quality corrected circular consensus sequence subreads set. Four databases, KEGG (Kyoto Encyclopedia of Genes and Genomes), COG (Clusters of Orthologous Groups), NR (Non-Redundant Protein Database databases), Swiss-Prot (Bairoch and Apweiler 1999), and GO (Gene Ontology), were used for general function annotation. Manual correction via alignments with *A. mirum* was performed for essential metabolism pathways construction. The antiSMASH (antibiotics & Secondary Metabolite Analysis Shell, http://antismash.secondarymetabolites.org/) was utilized to analyze the secondary metabolite gene clusters in *A. pretiosum* (Blin et al. 2019). The R package circlize (http://cran.r-project.org/web/packages/circlize/index.html) was adopted to draw the genome map (Gu et al. 2014).

### Construction of the pCRISPR–Cas9apre

Our CRISPR–Cas9 system was retrofitted by pCRISPR–Cas9 as a backbone to harbor both Cas9 and the sgRNA, which were driven by *tipAp* and *ermEp* promoter, respectively. The Cas9 encoding gene (4163 bp) was codon-optimized for *A. pretiosum* amplified from the Cas9 synthesized by Genscript (Nanjing, China, the sequence referenced pCRISPR–Cas9). Synthetic guide RNA (sgRNA) region (7158 bp), including the *Stu*I flanked *aac(3)IV* resistance selection cassettes and origin of transfer (*oriT*), were amplified from pCRISPR–Cas9 plasmid by primer pair Cas9backbone-F/R. Then, the two fragments were ligated to generate pCRISPR–Cas9ap (Additional file 1: Fig. S9). The pSG5 replicon was substituted by pIJ101 replicon amplified by primer pair YH7-F/R from pYH7. Primers used in this study are listed in Additional file 1: Table S5. Double fragment assembly was carried out by using One Step Cloning Kit (Vazyme, Nanjing, China).

### Construction of recombinant strains

#### Precise gene deletion using HDR

The genomic DNA of *A. pretiosum* subsp*. auranticum* was used as PCR template. The homologous arms and sgRNA guide sequences used for gene knockout were designed based on the genome sequence of strain L40. The pCRISPR–Cas9apreΔ*asm25*-sgRNA construction process as an example is illustrated in Additional file 1: Fig. S2. Briefly, two 1 kb homologous arms for *asm25* disruption were amplified, sequenced, and together cloned to *Stu*I-digested plasmid pCRISPR–Cas9apre by Gibson assembly to generate the pCRISPR–Cas9apreΔ*asm25*-sgRNA. The amplification primers are shown in Additional file 1: Table S5. The ApE software was used to search N20 targeting sequences of sgRNAs (http://ape-a-plasmid-editor.wikispaces.com). The relevant primers used to construct the functional sgRNA are shown in Additional file 1: Table S5. The sgRNA cassettes containing the 20-bp DNA region were cloned into the *Xma*JI/*Sna*BI site of pCRISPR–Cas9apreΔ*asm25* to generate pCRISPR–Cas9apreΔ*asm25*-sgRNA. The resulting plasmid was introduced into L40 from *E. coli* ET12567 (pUZ8002) through intergeneric conjugation. Conjugants were transferred to YMG plates with 1 μg/mL thiostrepton that induces Cas9 expression. The survivals were screened for the correct constructs by colony PCR and Sanger sequencing.

The iterative genome editing protocol is depicted in Fig. 4. The genotype confirmed edited colonies may use for the next round of editing. Before introducing new plasmids, the previous editing plasmid must be cured. The edited *A. pretiosum* mutants were cultured in TSBY without antibiotics for three rounds, 24 h per round. Subsequently, mutants were streaked on two different sets of agar plates with and without thiostrepton. Edited strains were selected by loss of thiostrepton resistance, which had already cured plasmid and were chosen for the next round of gene editing.

#### Construction of BDPs insertion plasmid

Two combined BDPs were spliced by overlap extension PCR, and cloned into *Stu*I-digested plasmid pCRISPR–Cas9apre with the relevant UHA (upper homologous arm) and DHA (down homologous arm) amplified from genomic DNA of L40 (Additional file 1: Fig. S7B). An sgRNA scaffold including gene specific 20-nt guide sequence TGCGGATCGTCACCGCCGCG was amplified from pCRISPR–Cas9apre, then cloned into p12J_K13CRISPR–Cas9apre at *Xma*JI/*Sna*BI by infusion cloning kit resulting plasmid p12J_K13sgCRISPR–Cas9apre (Additional file 1: Fig. S7C). Similarly, p12E_K13sgCRISPR–Cas9apre was generated according to the previous procedure. The resulting plasmids were, respectively, introduced into L40 using the method mentioned above and the recombinant strains were screened and validated.

Seamless assembly of multiple DNA fragments was carried out using NEB DNA Assembly Master Mix (New England Biolabs, Ipswich, MA). Restriction endonucleases were purchased from Thermo Fischer Scientific (Waltham, MA). All assay kits and enzymes were performed according to the manufacturers' recommendations.

### RNA isolation, cDNA synthesis and real-time quantitative PCR (RT-qPCR)

Total RNA was extracted using a bacterial RNA extraction kit (Jiangsu Cowin Biotech Co., Ltd., Taizhou, China). Total DNA was removed by DNase I, and reverse transcription was performed by cDNA Synthesis Kit (Jiangsu Cowin). The transcription of target genes was internally normalized to *16S rRNA* and determined by quantitative real-time PCR using a CFX96 Real-Time System (Bio-Rad, Richmond, CA). The relative level of genes expression was calculated using the 2^−ΔΔCt^ method (Livak and Schmittgen 2001). Three PCR replicate determinations were made for each transcription analysis.

### HPLC analysis of AP-3

The supernatant of the fermentation broth was extracted with triple volumes of ethyl acetate and evaporated to quantify AP-3 production. The residues were dissolved in methanol. The HPLC analysis of AP-3 was operated on Agilent series 1260 (Agilent Technologies, Inc., Santa Clara, CA) with a SinoChrom ODS-BP C18 column (4.6 mm × 250 mm, 5 μm, Elite, Dalian, China). The flow rate was 0.6 mL/min with 85% methanol and 15% water at 28 °C, and UV detection was set at 254 nm.

### Determination of concentrations of intracellular acyl-CoA esters

The concentrations of intracellular M-CoA and MM-CoA in the relevant strains at 48 h and 72 h of incubation were extracted following the method described by Lu et al. (2016) and determined by LC–MS/MS. For each time point, samples of 25 mL were harvested. One milliliter of culture was collected to quantify the dry cell weight. The liquid cell sample of remaining culture was transferred into a precooled tube containing quenching and extraction solution (acetonitrile/methanol/0.1% glacial acetate at a volume ratio of 45:45:10, − 20 °C). The extraction was performed by repetitive vortexing and cooling on ice for 15 min and centrifuged to collect the supernatant (12,000 rpm, 4 °C, 3 min). Samples were analyzed using an Atlantis BEH C18 (1.7 µm, 2.1 × 100 mm, Waters Co., Milford, MA) on a triple quadrupole MS (Waters). The mobile phase was acetonitrile with 50 mM ammonium hydrogen carbonate (solvent A), 0.1% ammonium hydroxide (solvent B), ddH_2_O (solvent C) and 0.1% ammonium hydroxide-acetonitrile (solvent D). Elution was performed as follows: 0–3 min 20% A 5% B 75% C, 3–3.5 min 20% A 10% B 30% C 40% D, 3.5–5 min 20% A 80% D, 5–7 min 20% A 5% B 75% C. Quantification was detected in the multiple reaction monitoring mode (MRM) with the *m*/*z* parent > *m*/*z* daughter (M-CoA 854 > 347, MM-CoA 868 > 361).

### Determination of concentrations of intracellular TAGs

TAGs were purified following the method described by Wang et al. (2020a) with some modifications. In brief, mycelia were collected by centrifugation at 4 °C, 12,000 rpm for 5 min. Mycelia were immediately submerged into liquid nitrogen and then lyophilized with a vacuum concentrator. Total lipids were extracted from 50 mg lyophilized cells by chloroform/methanol (2:1, v/v) in a water bath at 100 °C for 10 min. Subsequently, the mixture was shaken at 28 °C for 2 h at 250 rpm/min. TAGs samples were concentrated and dissolved with 1 mL of extraction solution. To determine the lipid compositions, TLC was carried out on silica gel 60 F_254_ plates (Arabolaza et al. 2008). Cu-phosphoric acid staining was used to visualize lipid fractions, and an imaging system (BG-gdsAUTO 720, Baygene Biotechnol Co., Ltd., Shanghai, China) was used to quantify bands of TAGs and phospholipids (PLs).

### Supplementary Information

**Additional file 1: Table S1.** Summary of genome editing results in *A. pretiosum*. **Table S2.** Biosynthetic gene clusters identified in the ATCC 31565 genome. **Table S3.** Putative PKS gene clusters and the predicted corresponding extender units. **Table S4.** Strains and plasmids used in this study. **Table S5.** Primers used in this study. **Figure** **S1.** Schematic post-PKS pathway in biosynthesis of AP-3 (adapted from Ning et al. 2017). **Figure S2.** Construction process of pCRISPR-Cas9apreΔ*asm25*-sgRNA for* asm25 *inactivation. **Figure S3.** Transformation efficiency of CRISPR-Cas9 system in *A. pretiosum* L40 with and without HDR. **Figure S4.** Schematic diagram of primary metabolism for ansamitocin production in *A. pretiosum* subsp. *auranticum* ATCC 31565. **Figure S5.** Location of T1PKS gene clusters and identification of gene cluster deletion mutants. **Figure S6.** Dry cell weight of gene cluster deletion mutants at the end of fermentation. **Figure S7.** Construction of bidirectional promoter knock-in mutant strains. **Figure S8.** Validation of bidirectional promoter knock-in mutant strains. **Figure S9.** Construction of pCRISPR-Cas9apre. **Figure S10.** Transcriptional analysis of *udpg* (A), AP-3 biosynthetic genes (B) and long-chain acyl-CoA synthetase genes (C) of strain MD15 at day 3 of fermentation. **Figure S11.** Transcriptional profiles of fatty acyl-CoA synthetase genes in L40 (dark) and MD04 (gray).

## Data Availability

All data generated or analyzed during this study are included in this article.

## Abbreviations

AP-3: Ansamitocin P-3
HDR: Homology-directed repair
BDPs: Bidirectional promoters
GC: Guanine-cytosine
M-CoA: Malonyl-CoA
MM-CoA: Methylmalonyl-CoA
MM-ACP: Methoxymalonyl-acyl carrier protein
AHBA: 3-Amino-5-hydroxybenzoic acid
1,3-BPG: 1,3-Bisphosphoglycerate
PP: Pentose phosphate
KEGG: Kyoto Encyclopedia of Genes and Genomes
COG: Clusters of Orthologous Groups
NR: Non-redundant protein database
GO: Gene ontology
antiSMASH: antibiotics & Secondary Metabolite Analysis SHell
sgRNA: Synthetic guide RNA
UHA: Upper homologous arm
DHA: Down homologous arm
RT-qPCR: Real-time quantitative PCR
PNDs: N-Demethylansamitocins
PND-3: N-Demethyl-AP-3
TAGs: Triacylglycerols
SD: Standard deviation
