# Supplementary material for: Two strategies to improve the supply of PKS extender units for ansamitocin P-3 biosynthesis by CRISPR–Cas9
Source: Bioresour Bioprocess. 2022 Aug 29;9(1):90. doi: 10.1186/s40643-022-00583-7 (PMC10991131; doi:10.1186/s40643-022-00583-7)
Supplement: Supplementary file 1 — Additional file 1: Table S1. Summary of genome editing results in A. pretiosum. Table S2. Biosynthetic gene clusters identified in the ATCC 31565 genome. Table S3. Putative PKS gene clusters and the predicted corresponding extender units. Table S4. Strains and plasmids used in this study. Table S5. Primers used in this study. Figure S1. Schematic post-PKS pathway in biosynthesis of AP-3 (adapted from Ning et al. 2017). Figure S2. Construction process of pCRISPR-Cas9apreΔasm25-sgRNA for asm25 inactivation. Figure S3. Transformation efficiency of CRISPR-Cas9 system in A. pretiosum L40 with and without HDR. Figure S4. Schematic diagram of primary metabolism for ansamitocin production in A. pretiosum subsp. auranticum ATCC 31565. Figure S5. Location of T1PKS gene clusters and identification of gene cluster deletion mutants. Figure S6. Dry cell weight of gene cluster deletion mutants at the end of fermentation. Figure S7. Construction of bidirectional promoter knock-in mutant strains. Figure S8. Validation of bidirectional promoter knock-in mutant strains. Figure S9. Construction of pCRISPR-Cas9apre. Figure S10. Transcriptional analysis of udpg (A), AP-3 biosynthetic genes (B) and long-chain acyl-CoA synthetase genes (C) of strain MD15 at day 3 of fermentation. Figure S11. Transcriptional profiles of fatty acyl-CoA synthetase genes in L40 (dark) and MD04 (gray). [file 40643_2022_583_MOESM1_ESM.docx]

Additional file

**Two strategies to improve the supply of PKS extender units for ansamitocin P-3 biosynthesis by CRISPR-Cas9**

Siyu Guo^1^, Xueyuan Sun^1^, Ruihua Li^1^, Tianyao Zhang^1^, Fengxian Hu^1^, Feng Liu^1^*, Qiang Hua^1, 2^*

Affiliations

1 State Key Laboratory of Bioreactor Engineering, East China University of Science and Technology, 130 Meilong Road, Shanghai 200237, China

2 Shanghai Collaborative Innovation Center for Biomanufacturing Technology, 130 Meilong Road, Shanghai 200237, China

* Corresponding author: Feng Liu

E-mail addresses: fengliu@ecust.edu.cn

* Corresponding author: Qiang Hua

E-mail addresses: qhua@ecust.edu.cn

Phone: +86-21-64250972

Table S1 Summary of genome editing results in *A. pretiosum*

| **Targets** | **Host cell** | **N20 target seq** | **PAM** | **Deletion size** | **Editing efficiency*** |
| --- | --- | --- | --- | --- | --- |
| *asm25* | L40 | cagccccagcgcggcgaccc | tGG | 61 bp | 19/24 |
| T1PKS-15 | L40 | gtcgtcgccgaaccgccggg | gGG | 881 bp | 1/1 |
|  | MD01 | cgacgacgtggtgatcgccg | cGG |  | 3/4 |
| T1PKS-16 | L40 | gctgcgcgcggcgtaccggg | cGG | 35,774 bp | 4/8 |
|  | MD02 | gatggtgccggaactggcgg | cGG |  | 2/4 |
| T1PKS-18 | L40 | gaccggttctggacgcccgg | cGG | 49,948 bp | 1/4 |
|  | MD01 | gcacccggtcctcggcccgg | cGG |  | 3/10 |
| NRPS-25 | L40 | gccgagtgcgagctgccgga | gGG | 53,335 bp | 3/4 |
| T1PKS/NRPS-5 | L40 | cctcacctccgtgttccacg | cGG | 61,577 bp | 1/3 |
|  | MD02 |  |  |  | 1/1 |

Note: *, number of positive mutant strains obtained from all single colonies observed on plates.

Table S2 Biosynthetic gene clusters identified in the ATCC 31565 genome

| **Gene cluster** | **Type** | **Location(bp)** | **Gene Order** |
| --- | --- | --- | --- |
| REGION 1 | Terpene | 1067413-1087584 | 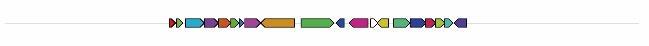 |
| REGION 2 | bacteriocin | 1695961-1706761 | 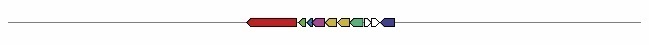 |
| REGION 3 | Indole | 2324861-2345982 | 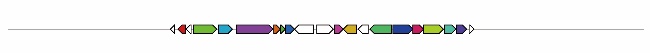 |
| REGION 4 | T1pks-Nrps | 2792267-2836794 | 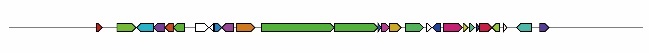 |
| REGION 5 | T1pks-Nrps | 3025815-3183852 | 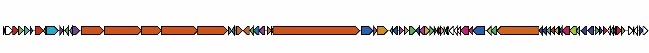 |
| REGION 6 | Nucleoside | 3240804-3261772 | 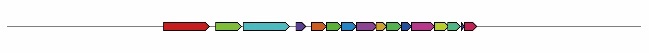 |
| REGION 7 | T1pks-Nrps | 3509071-3588778 | 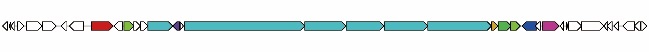 |
| REGION 8 | Other | 3670964-3710653 | 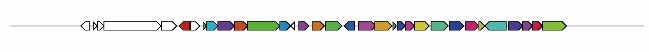 |
| **REGION 9** | **T1pks** | **3734880-3831692** | **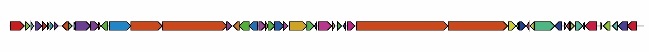** |
| REGION 10 | Lassopeptide | 3890415-3912878 | 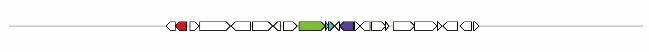 |
| REGION 11 | T1pks-Nrps | 3922947-4010591 | 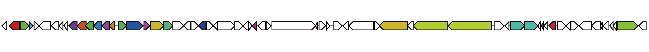 |
| REGION 12 | Nrps-Otherks | 4184804-4332934 | 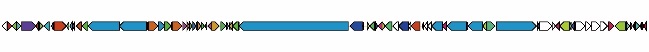 |
| REGION 13 | Terpene | 4452203-4471712 | 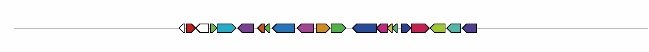 |
| REGION 14 | Thiopepitde | 4496783-4526236 | 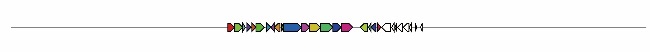 |
| REGION 15 | T1pks | 4532111-4572806 | 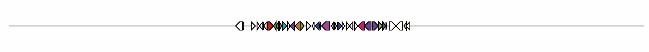 |
| REGION 16 | T1pks | 4681606-4737212 | 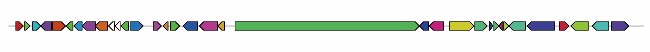 |
| REGION 17 | T1pks | 4770498-4815318 | 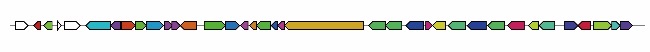 |
| REGION 18 | T1pks | 5101692-5162844 | 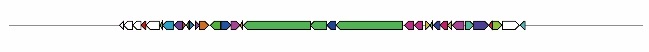 |
| REGION 19 | Terpene | 5173202-5194234 | 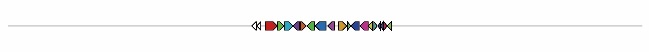 |
| REGION 20 | Nrps | 5222058-5315000 | 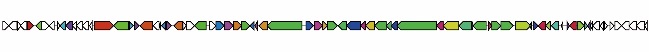 |
| REGION 21 | Nrps | 5352487-5395473 | 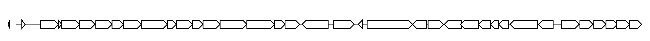 |
| REGION 22 | T1pks-Nrps | 5395516-5464032 | 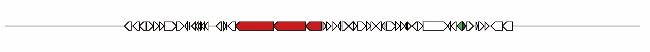 |
| REGION 23 | T1pks | 5498004-5556793 | 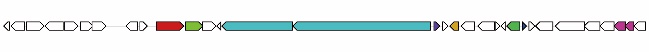 |
| REGION 24 | Oligosaccharide | 5768194-581177 | 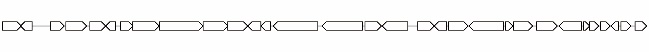 |
| REGION 25 | Nrps | 5870734-5924068 | 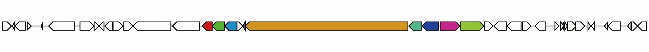 |
| REGION 26 | Terpene | 6030239-6051303 | 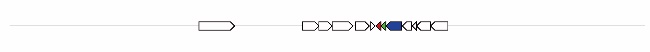 |
| REGION 27 | Lanthipeptide | 6214120-6237188 | 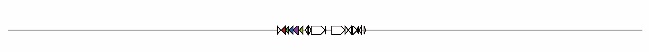 |

Table S3 Putative PKS gene clusters and the predicted corresponding extender units

| **Region** | **Gene cluster type** | **Location** | **M-CoA** | **MM-CoA** |
| --- | --- | --- | --- | --- |
| T1PKS/NRPS-5 | Type I PKS/NRPS | 3,025,815-3,183,852 | 7 | 3 |
| T1PKS-11 | Type I PKS/NRPS | 3,922,947-4,010,591 | 1 | NA |
| T1PKS-15 | Type I PKS | 4,532,111-4,572,806 | 1 | NA |
| T1PKS-16 | Type I PKS | 4,681,606-4,737,212 | 2 | NA |
| T1PKS-17 | Type I PKS | 4,770,498-4,815,318 | 1 | NA |
| T1PKS-18 | Type I PKS | 5,101,692-5,162,844 | 1 | 1 |
| T1PKS/NRPS-22 | Type I PKS/NRPS | 5,395,516-5,464,032 | 1 | 1 |
| T1PKS-23 | Type I PKS | 5,498,004-5,556,793 | 3 | 1 |
| **Ansamitocin gene cluster** | **Type I PKS** | **3,734,880-3,831,692** | **3** | **3** |

Table S4 Strains and plasmids used in this study

| **Strains** | **Relevant properties** | **Source or reference** |
| --- | --- | --- |
| ***A. pretiosum* subsp. *auranticum*** |  |  |
| ATCC 31565 | Wild-type producer for ansamitocin P-3 | ATCC |
| L40 | ARTP mutant derived from ATCC 31565 | (Li et al. 2021) |
| MD01 | L40 with *asm25* deletion | This work |
| MD02 | L40 with T1PKS-15 deletion | This work |
| MD03 | L40 with T1PKS-16 deletion | This work |
| MD04 | L40 with T1PKS-18 deletion | This work |
| MD05 | L40 with T1PKS/NRPS-5 deletion | This work |
| MD06 | L40 with NRPS-25 deletion | This work |
| MD15 | MD02 with T1PKS/NRPS-5 deletion | This work |
| BDP-ek | L40 with *ermEp*-kasOp** inserted in spacer of *asm12*-*asm13* | This work |
| BDP-jk | L40 with *j23119p*-kasOp** inserted in spacer of *asm12*-*asm13* | This work |
| ***E. coli*** |  |  |
| DH10B | Cloning host | Invitrogen |
| ET12567/pUZ8002 | For intergeneric conjugation | (Paget et al. 1999) |
| **Plasmids** |  |  |
| pCRISPR-Cas9 | *ori(ColE1), rep(pSG5), oriT(RK2), aac(3)IV, tipAp*, tsr* | (Tong et al. 2015) |
| pYH7 | *rep(pIJ101)*, *ori(ColE1)*, *oriT*, *aac(3)IV* | (Sun et al. 2009) |
| pCRISPR-Cas9ap | pCRISPR-Cas9 derivative, codon-optimized cas9 towards *A. pretiosum* | This work |
| pCRISPR-Cas9Δ*asm25* | pCRISPR-Cas9 carries sgRNA for Δ*asm25* and homologous recombination template with *Φ*C31 *attB* | This work |
| pCRISPR-Cas9apΔ*asm25* | pCRISPR-Cas9ap carries sgRNA for Δ*asm25* and homologous recombination template with *Φ*C31 *attB* | This work |
| pCRISPR-Cas9apre | pCRISPR-Cas9 derivative, *rep(pIJ101),* codon-optimized cas9 towards *A. pretiosum*, *Xma*JI-*Sna*BI sgRNA cloning cassette | This work |
| pCRISPR-Cas9apreΔ*asm25* | pCRISPR-Cas9apre carries sgRNA for Δasm25 and homologous recombination template with *Φ*C31 *attB* | This work |
| pCRISPR-Cas9apreΔT1PKS/NRPS-5 | pCRISPR-Cas9apre derivative, for T1PKS/NRPS-5 deletion | This work |
| pCRISPR-Cas9apreΔT1PKS-15 | pCRISPR-Cas9apre derivative, for T1PKS-15 deletion | This work |
| pCRISPR-Cas9apreΔT1PKS-16 | pCRISPR-Cas9apre derivative, for T1PKS-16 deletion | This work |
| pCRISPR-Cas9apreΔT1PKS-18 | pCRISPR-Cas9apre derivative, for T1PKS-18 deletion | This work |
| pCRISPR-Cas9apreΔNRPS-25 | pCRISPR-Cas9apre derivative, for NRPS-25 deletion | This work |
| p12E_K13sgCRISPR-Cas9apre | pCRISPR-Cas9apre derivative, for *ermEp*-*kasOp* insertion in spacer of asm12-asm13 | This work |
| p12J_K13sgCRISPR-Cas9apre | pCRISPR-Cas9apre derivative, for *j23119p-kasOp* insertion in spacer of *asm12*-*asm13* | This work |

Table S5 Primers used in this study

| **Primers** | **Sequences (from 5′ to 3′)^a, b^** |
| --- | --- |
| **Primers for the construction of the pCRISPR-Cas9apre** | |
| olCas9ap-f | CGGCGTCAGAGAAGGGAGCGGACATATGGACAAGAAGTACTCCATCGGCCTGGAC, *Nde*I site underlined |
| olCas9ap-r | GCTCGGTACCCGGGGATCCTCTAGAAAGCTTTCAGTCGCCGCCCAGCTGCGACAG, *Hind*III site underlined |
| Cas9backbone-F | ATGTCCGCTCCCTTCTCTGACGCCGTCCACGCTGCCTCCTCACGTGA |
| Cas9backbone-R | AAGCTTTCTAGAGGATCCCCGGGTACCGAGCTCGAATTCC |
| YH7-F | TCGCTCGCCGACACACGCACTGACCGCACGTCAAAGCCCC |
| YH7-R | TTTCCAGACTTTACGAAACACGGAAACCGAAGACCATTCATGT |
| **Primers for the construction and identification of deletion mutants** | |
| olΔasm25UHA-f | ATCTCGTCGAAGGCACTAGAAGGCCTCTCCCCGGCGGGCGTGTCCT |
| olΔasm25UHA_ΦC31-r | GGGGAGCCCAAGGGCACGCCCTGGCACCCGCACCGCCGCCGATCTGGCGGACGCCTG |
| olΔasm25DHA_ΦC31-f | GGCGTGCCCTTGGGCTCCCCGGGCGCGTACTCCACCCTGGTCGCCGAACAGCGGGGC |
| olΔasm25DHA-r | CGCGGTCGATCCCCGCATATAGGCCGCGGAGCTGTTCGAGTTCA |
| T1PKS/NRPS-5UHA-f | CGGGATCTCGTCGAAGGCACTAGAGTGCTCGACAACGAACTCGGCATC |
| T1PKS/NRPS-5UHA-r | CGTTCACACTGCTCGGTCATAACTACTAGTACCGCCATGACGACCTTGATCAGC, *Bcu*I site underlined |
| T1PKS/NRPS-5DHA-f | GCTGATCAAGGTCGTCATGGCGGTACTAGTAGTTATGACCGAGCAGTGTGAACG, *Bcu*I site underlined |
| T1PKS/NRPS-5DHA-r | CCCGCGCGGTCGATCCCCGCATATCATCCACCGGTTGCGCAGGACCCAC |
| T1PKS-15UHA-f | CGGGATCTCGTCGAAGGCACTAGAAATTCTGGGCGCCTTGCAACGTCTCGCG |
| T1PKS-15UHA-r | GGGGAGCCCAAGGGCACGCCCTGGCACCCGCACCGAACCACGCCTTCCACTCCCCGCTGATGG |
| T1PKS-15DHA-f | GGCGTGCCCTTGGGCTCCCCGGGCGCGTACTCCACCAAAGCCCGAGCCCCGGCAGGTCGAT |
| T1PKS-15DHA-r | CCCGCGCGGTCGATCCCCGCATATCTCGGAGCTGACCTGGTCCGTGGTC |
| T1PKS-16DHA-f | ACAAGTGAGATATCGAATTCGTATCACGGCCGGACCGACGGTGTCCT |
| T1PKS-16DHA-r | CGCGCGGTCGATCCCCGCATATTGTTCAGCGCCACCTACCTGGACC |
| T1PKS-16UHA-f | ATCTCGTCGAAGGCACTAGAACTCCAACGCCTACGTCGCGAGCG |
| T1PKS-16UHA-r | GCAGCCCAAGCTTGGCACTGGCGAAACCCGTGCGCACCAACGTCTG |
| T1PKS-18UHA-f | ATCTCGTCGAAGGCACTAGATCGGGTGCAGCTGGACGGGCTTCT |
| T1PKS-18UHA-r | CGGTGAACGAGAACGTCTTGCCGGCCGACCCGATGCGAGCCGCCGGCTGACGAGTCACC |
| T1PKS-18DHA-f | CCGGCAAGACGTTCTCGTTCACCGGCTGGAAGGTCTCGGCGGCGATGTCCGCGGTCGCC |
| T1PKS-18DHA-r | CGCGGTCGATCCCCGCATATTGCTGGACGACCTGCCGAACCTGC |
| NRPS-25UHA-f | TGCGGGATCTCGTCGAAGGCACTAGACGAGGACGTGGACGCGATGCGCAG |
| NRPS-25UHA-r | TTTTATCACTGCTTTGGCATGGTGATGGGGAAACTAGTGGAAAGCTACCGAGGACCCCCGACA |
| NRPS-25DHA-r | CCGCGCGGTCGATCCCCGCATATCGCAGATCATGTTGCGACGCAAGGGA |
| NRPS-25DHA-f | CCCATCACCATGCCAAAGCAGTGATAAAAGGGCACCGGGCGTTCCATGCCCTGTCCGGGTGTC |
| Δasm25test-f | CAGCTCGGACCGCATCCCCGACC |
| Δasm25test-r | CGACCCTGCTGTTCGACCACCCGACG |
| ΔT1PKS/NRPS-5yz-f | ACCTGCGCCGCATGGAGCGGC |
| ΔT1PKS/NRPS-5yz-r | GTGGCCTTCCAGGTGGTGTTCTGGCC |
| ΔT1PKS-15check-f | ATGACGTGATCCCACAACGCCGTGG |
| ΔT1PKS-15check-r | TTGTTGTGATCTCTGGTGAGGGAGTT |
| ΔT1PKS-16check-f | CTGTGAAAAAGCCCCGCCGC |
| ΔT1PKS-16check-r | CCATGCCGGTGATTAGCGCGGT |
| ΔT1PKS-18check-f | ACCTGCGCCGCATGGAGCGGC |
| ΔT1PKS-18check-r | GTGGCCTTCCAGGTGGTGTTCTGGCC |
| ΔNRPS-25check-f | TGTTGATATCGGAGCAGAGCTGAC |
| ΔNRPS-25check-r | AACGATCTGCGCATCCTGCCTGCG |
| **Primers for the construction and identification of BDP-inserted mutants** | |
| BDP-a | CAGCGTGCAGGACTGGGGGAGTTACTGTCCACAACCGACCCG |
| BDP-b | ACCCGCGCGGTCGATCCCCGCATATTTGTGGCCGCGCACCAGGAC |
| BDP-c | TGCGGGATCTCGTCGAAGGCACTAGACGGCCGACCGTGCCCGCGA |
| BDP-d | GGAACCGGCTTCGCGCCGC |
| BDP-e | TGGAGCTGGGGACCGGGTCGGTTGTGGACAGTAACTCCCCCAGTCCTGCA |
| BDP-f | CCGTCGGGCGGCGCGAAGCCGGTTCCGCTAGCATTATACCTAGGACTGAG |
| BDP-g | CCGGCCGTCGGGCGGCGCGAAGCCGGTTCCTGGATCCTACCAACCGGCAC |
| BDPcheck-f | AGCATCGACTCGCCGATGTGGAAG |
| BDPcheck-r | TCCGGGTCCACCAGGAGCACGGGC |
| **Primers for amplification of sgRNA cloning cassette** | |
| asm25-sg-F | TGGTAGGATCGACGGCCTAGG **CAGCCCCAGCGCGGCGACCC** GTTTTAGAGCTAGAAATAGC, *Xma*JI site underlined |
| T1PKS/NRPS-5-sg-F | TGGTAGGATCGACGGCCTAGG **CCTCACCTCCGTGTTCCACG** GTTTTAGAGCTAGAAATAGC, XmaJI site underlined |
| T1PKS-15-sg1-F | TGGTAGGATCGACGGCCTAGG **GTCGTCGCCGAACCGCCGGG** GTTTTAGAGCTAGAAATAGC, *Xma*JI site underlined |
| T1PKS-15-sg2-F | TGGTAGGATCGACGGCCTAGG **CGACGACGTGGTGATCGCCG** GTTTTAGAGCTAGAAATAGC, *Xma*JI site underlined |
| T1PKS-16-sg1-F | TGGTAGGATCGACGGCCTAGG **GCTGCGCGCGGCGTACCGGG** GTTTTAGAGCTAGAAATAGC, *Xma*JI site underlined |
| T1PKS-16-sg2-F | TGGTAGGATCGACGGCCTAGG **GATGGTGCCGGAACTGGCGG** GTTTTAGAGCTAGAAATAGC, *Xma*JI site underlined |
| T1PKS-18-sg1-F | TGGTAGGATCGACGGCCTAGG **GACCGGTTCTGGACGCCCGG** GTTTTAGAGCTAGAAATAGC, *Xma*JI site underlined |
| T1PKS-18-sg2-F | TGGTAGGATCGACGGCCTAGG **GCACCCGGTCCTCGGCCCGG** GTTTTAGAGCTAGAAATAGC, *Xma*JI site underlined |
| NRPS-25-sg-F | TGGTAGGATCGACGGCCTAGG **GCCGAGTGCGAGCTGCCGGA** GTTTTAGAGCTAGAAATAGC, *Xma*JI site underlined |
| BDPsg7-F | TGGTAGGATCGACGGCCTAGG **GTGGGCGGTTCGTTACCCGG** GTTTTAGAGCTAGAAATAGC, *Xma*JI site underlined |
| BDPsg8-F | TGGTAGGATCGACGGCCTAGG **TGCGGATCGTCACCGCCGCG** GTTTTAGAGCTAGAAATAGC, *Xma*JI site underlined |
| sgRNA-R | TCAGCAGTCCCCGGAACATCGTAGCTGACGCCTACGTAAAAAAAGCACCGACTCGGTGCC, *Sna*BI site underlined |
| **Primers used in RT-qPCR analysis** | |
| asmC-RT-F | TAGAGCCCGGCCAGGTCCCA |
| asmC-RT-R | ACGAGAAGCTGGTCGACTAC |
| T1PKS/NRPS-5-RT-F | ATGAGCCAGCCCGAGAACGCCGAGC |
| T1PKS/NRPS-5-RT-R | GCTCGGCGTTCTCGGGCTGGCTCAT |
| T1PKS-11-RT-F | CCGACCACGACCGCACCT |
| T1PKS-11-RT-R | TCACCGGCACCTCGACGC |
| T1PKS-15-RT-F | CTGCCCTGATCCGGTCCATTC |
| T1PKS-15-RT-R | GCTCGCCGATGCCGAAGAA |
| T1PKS-16-RT-F | GTCTGGGAGGCCCTGGAGGA |
| T1PKS-16-RT-R | GCGACCAGCGACGACGACT |
| T1PKS-17-RT-F | GCGGAGCTGTGGGAGAACGT |
| T1PKS-17-RT-R | GAGCCAGTGGGTGAGGTCGGT |
| T1PKS-18-RT-F | CCGGCACAGCAGCACAGC |
| T1PKS-18-RT-R | TGGTCCCGTGGGTGGTGTC |
| T1PKS/NRPS-22-RT-F | TTGTCGGACGAGGCGAGGCT |
| T1PKS/NRPS-22-RT-R | CGGATCGGGGTCGTAGGTGA |
| T1PKS-23-RT-F | ACGCCATCACCGACGTTCCC |
| T1PKS-23-RT-R | GCGGATGCTGCCGACGAA |
| NRPS-25-RT-F | GTCGTCACGCACCGGGAGTT |
| NRPS-25-RT-R | CCCGCATCACGAGCAGCAA |
| 16s rRNA-RT-F | CAGAAGAAGCACCGGCTAAC |
| 16s rRNA-RT-R | TTAAGCCCCAAGTTTTCACG |
| udpg-RT-F | CGACACCGACGACGACGA |
| udpg-RT-R | ACCGCAGCCTTGAGGAAACC |
| asm7-RT-F | TCCCCGCTGGAGCTGGTG |
| asm7-RT-R | CCTCGTCGAACGGGTCGC |
| asm10-RT-R | TCTGCCCGAGCAGCCCCGGAT |
| asm10-RT-F | ACGAGAGCTGGACGTCGCTG |
| asm11-RT-R | CGTCGGCGATCAGGAAGTGC |
| asm11-RT-F | CGTGGAGCTGCTGGACTGCG |
| asm12-RT-F | AGGAGTTCCCCCGCTTCCAC |
| asm12-RT-R | TCCTCCAGCAGCCCGACGAC |
| asm13-RT-F | CTCCAACACCTCCTCCATCC |
| asm13-RT-R | GTTGATCATCGGGTGCAGC |
| asm14-RT-F | CGGCCTGTCGTCGCTGTT |
| asm14-RT-R | GCACCGTGCGGAAGTTGT |
| asm15-RT-F | TGCCCTCGATGATCTCCATGA |
| asm15-RT-R | GCCACCGTGCTCGCCAAG |
| asm16-RT-F | ATCCGCAGCACCAGTCCCA |
| asm16-RT-R | TTCAGCCCCGCCGTCGTCA |
| asm17-RT-F | GACGGGCCAGGGTCATGC |
| asm17-RT-R | TGGACCCCGACACCGAGG |
| asm34-RT-F | TCGCCCTGCTGGAACTGC |
| asm34-RT-R | GGTCGGGGAGGGTGAGGC |
| fadD_0652-RT-F | TCCTCAGCATGATCGACTCCGGGC |
| fadD_0652-RT-R | TAGAGCATCGGCAGGGTCAGGAAG |
| fadD_1082-RT-F | TACCTGCGCTGGCGCGTCAC |
| fadD_1082-RT-R | CTGTTGAGCAGGACCACGTCGGCC |
| fadD_3288-RT-F | TGGCAGAGCTGACCGCACGG |
| fadD_3288-RT-R | AGGTCTCGGCGATGCCCGCG |
| fadD_3797-RT-F | TACGAGTTCCTGCACGACGA |
| fadD_3797-RT-R | AAGCGCTCCGTCACCCGCTC |
| fadD_4614-RT-F | GCAGGTCGAGTGGATCCTC |
| fadD_4614-RT-R | GGAGGTGTAGACGATCGTGG |
| fadD_4816-RT-F | TTCGCGCCGTGGAGCGAGGTG |
| fadD_4816-RT-R | GTTGGGGCGGCCCTTGAAGTC |
| **Primers used for RT-PCR of the *asm* cluster transcript assay** | |
| asmA-B(rt)-F | AACAGGTCCGCCAGGTCCC |
| asmA-B(rt)-R | GCCGGGTTCGACTCGATCAC |
| asmC-D(rt)-F | GGGCTGATGCCGAAGAAGG |
| asmC-D(rt)-R | ACGCCGAGGACCCCACGT |
| asm2-3-4(rt)-F | GTGCAGCACCAGCACCCG |
| asm2-3-4(rt)-R | TCGACGCCCACGTCCTCG |
| asm4-5(rt)-F | GCTCGCAGCCGACCAGGAACAT |
| asm4-5(rt)-R | CCAGGACCGTGCTGAAGAACCC |
| asm8-C(rt)-F | GCCGTCCACCAGCTCCCACA |
| asm8-C(rt)-R | GTTCGTCACCCCGAGCACC |
| asmD-9(rt)-F | AAGGTCGCGTCCACGTCCAG |
| asmD-9(rt)-R | ACCGGGTTCACCTCGCTCAC |
| asm11-12(rt)-F | CATCACCGACACCAGCCTGCCC |
| asm11-12(rt)-R | TGGTCGACGTTCTCGCCGTT |
| asm14-15(rt)-F | TGTCGTCGCTGTTCGCGATGGAG |
| asm14-15(rt)-R | GTCAGCTCGCCGTTGTCCAG |
| asm15-16(rt)-R | ACTTGACGCCCCAGCCGATCTCG |
| asm15-16(rt)-F | GTCTCCACCAGGGTGTGCGAGC |
| asm16-17(rt)-R | ACGAGCACCTCGATCCGGTCCGC |
| asm16-17(rt)-F | GAGGCGCGTGCCAGCGGGAACC |
| asm18-19(rt)-F | ACCCGCTGAGCACGAAGAAC |
| asm18-19(rt)-R | CGTCGCCTACCTGACCATCCC |
| asm34-35(rt)-F | CCCGATGCCCTGGACGAA |
| asm34-35(rt)-R | CCTGGAACGCCTCACCCTC |

Note: ^a^, The overlap sequence for DNA assembly is shown in red. ^b^, The N20 target sequences of sgRNA are shown in bold.


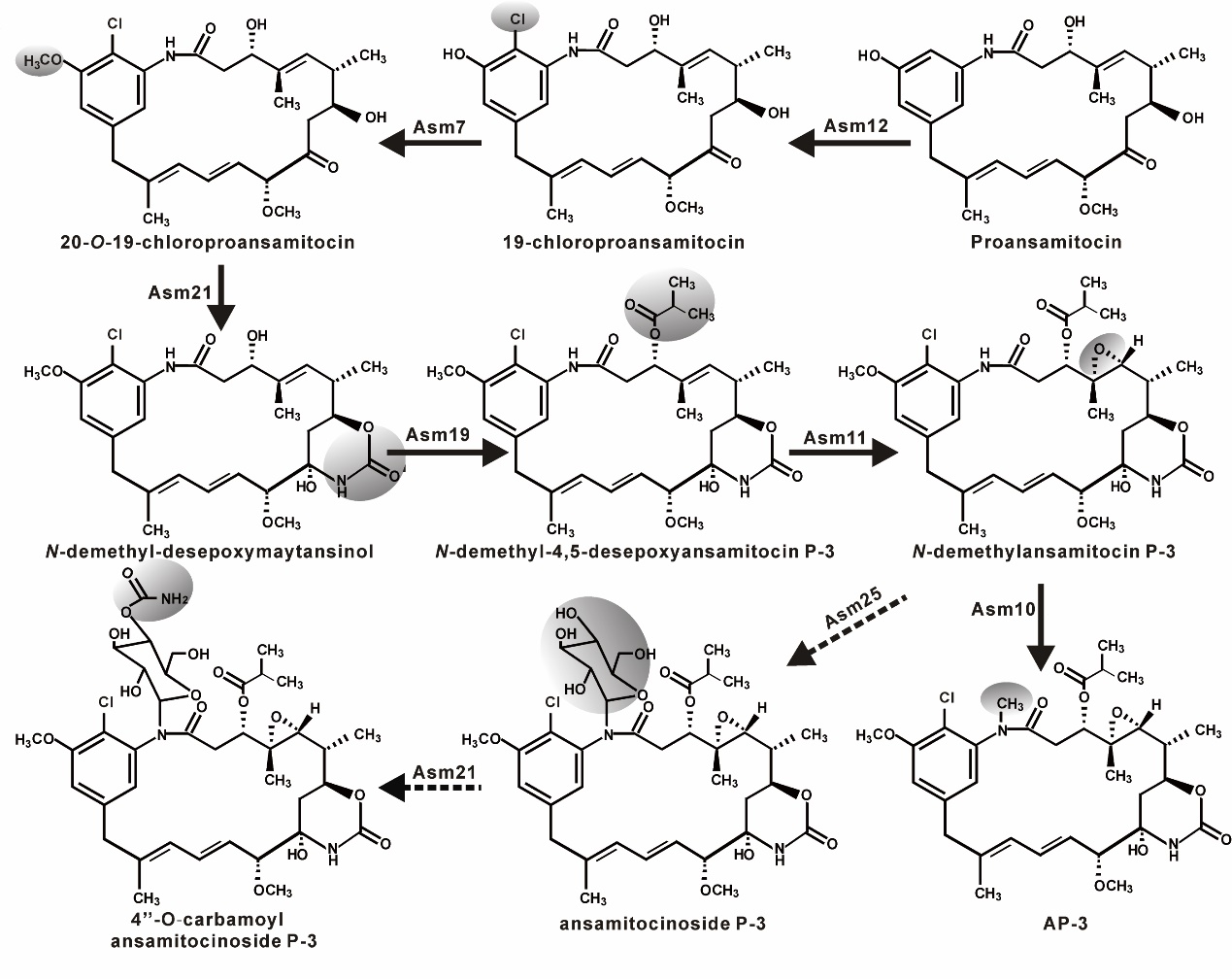
**Fig. S1 Schematic post-PKS pathway in biosynthesis of AP-3 (adapted from Ning et al. 2017)**


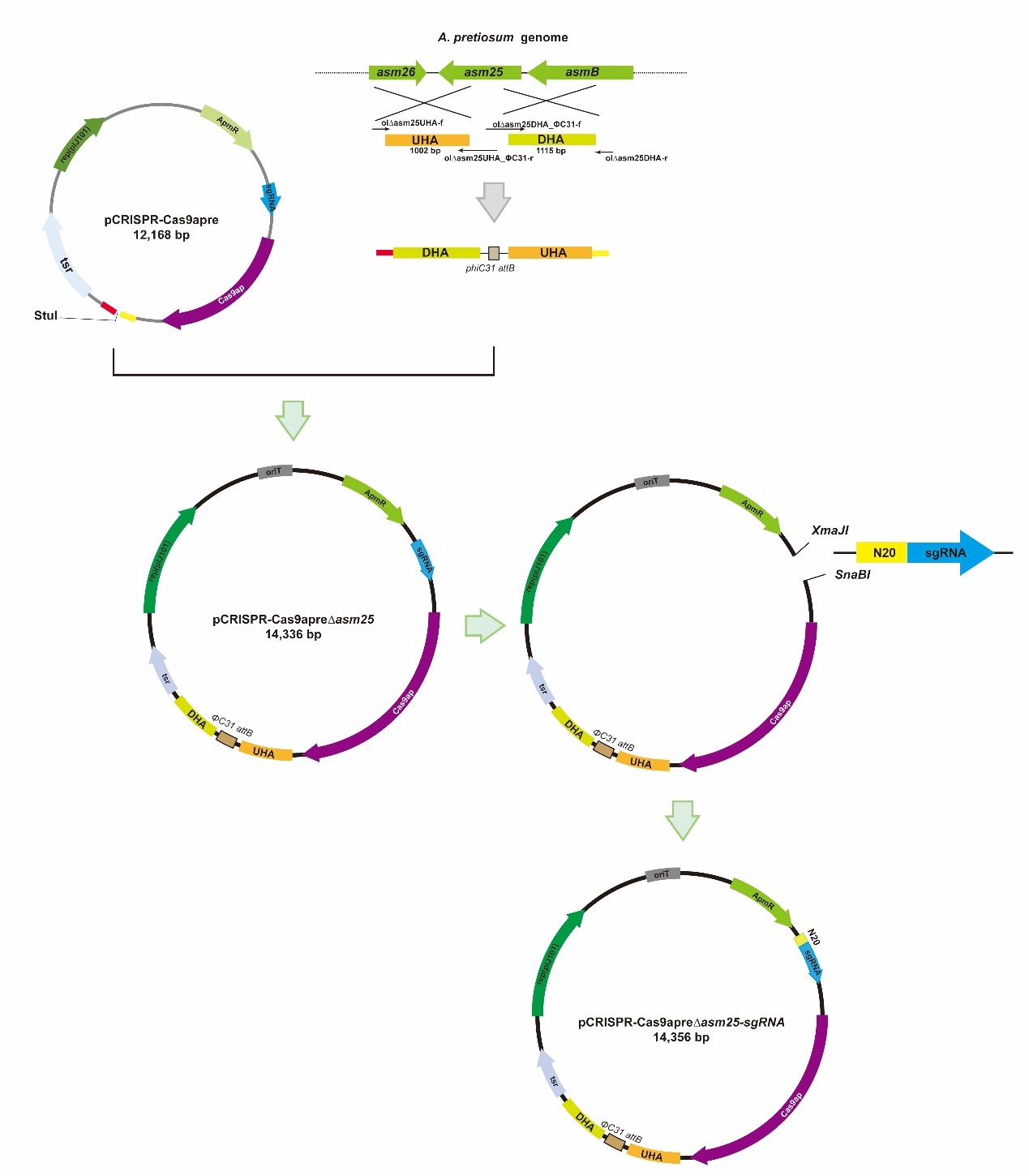


Fig. S2 Construction process of pCRISPR-Cas9apreΔ*asm25*-sgRNA for *asm25* inactivation


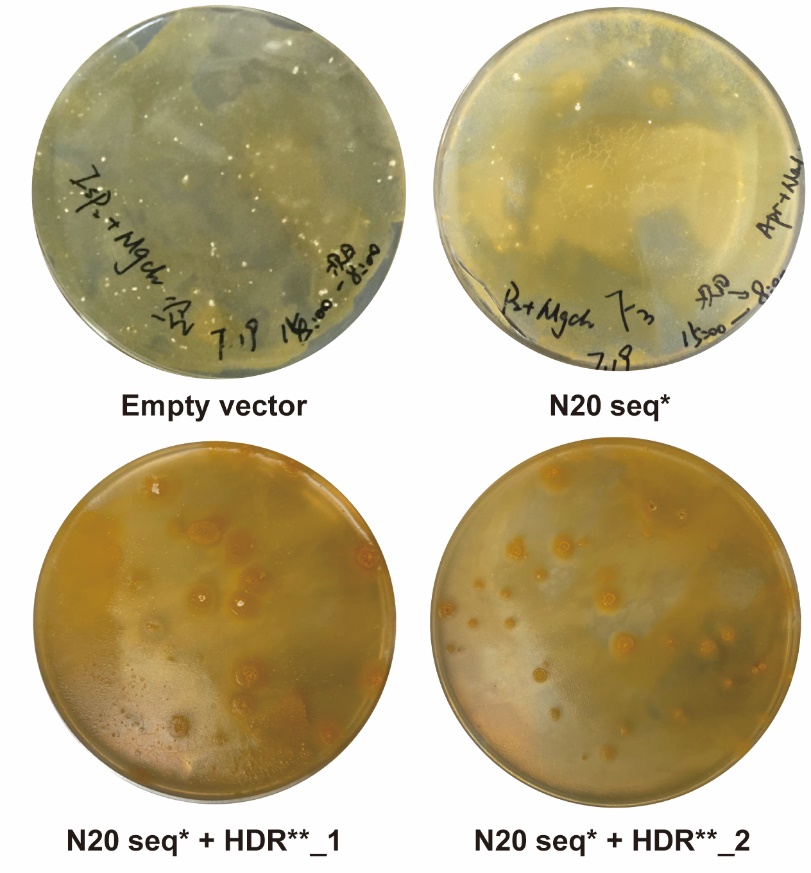


Fig. S3 Transformation efficiency of CRISPR-Cas9 system in *A. pretiosum* L40 with and without HDR

*, Containing N20 sgRNA sequence; **, Containing the template of HDR


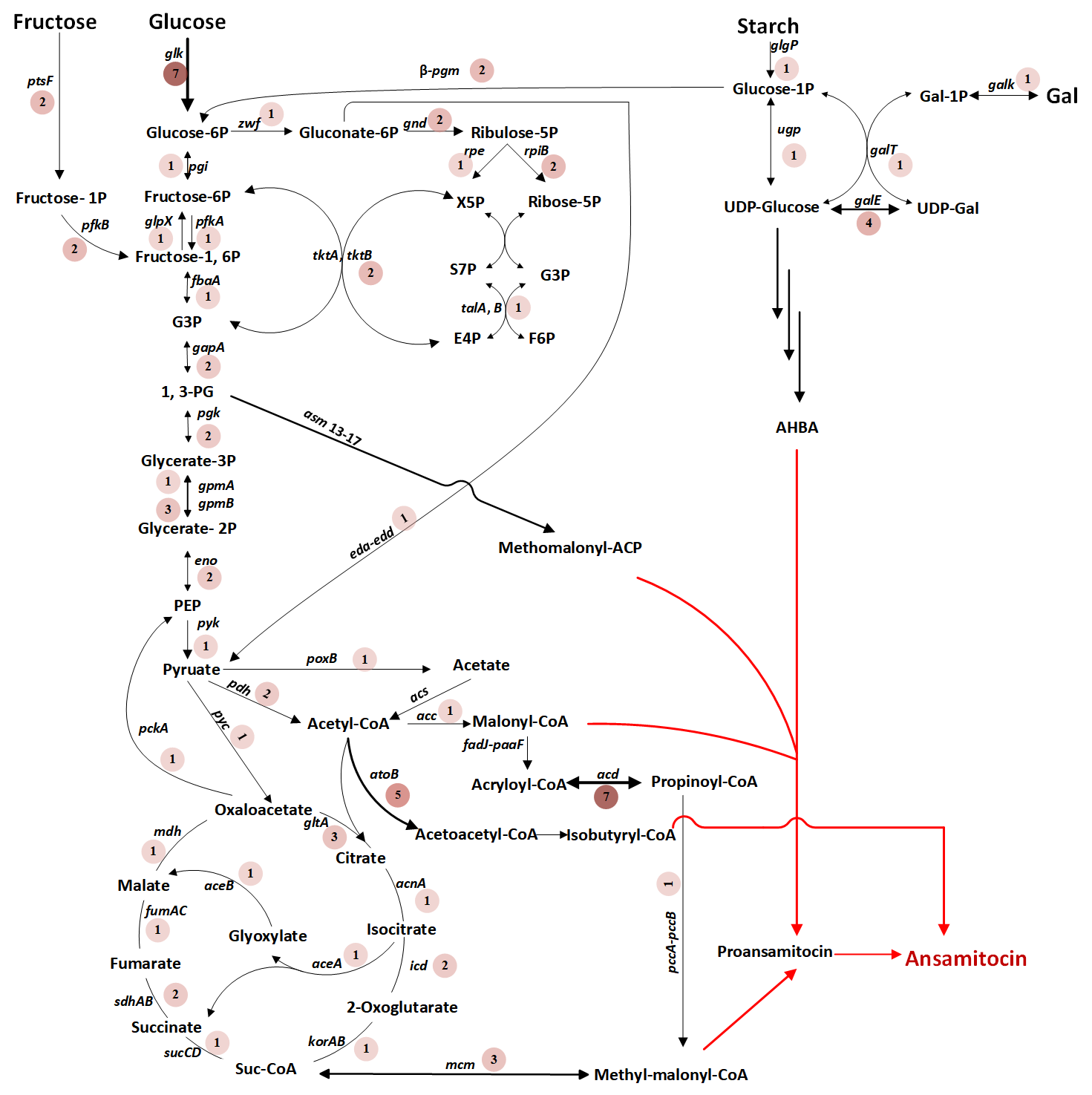


Fig. S4 Schematic diagram of primary metabolism for ansamitocin production in *A. pretiosum* subsp. *auranticum* ATCC 31565

The number in circular patterns indicates the gene copy number. Red arrows represent precursors directly related to ansamitocin biosynthesis.


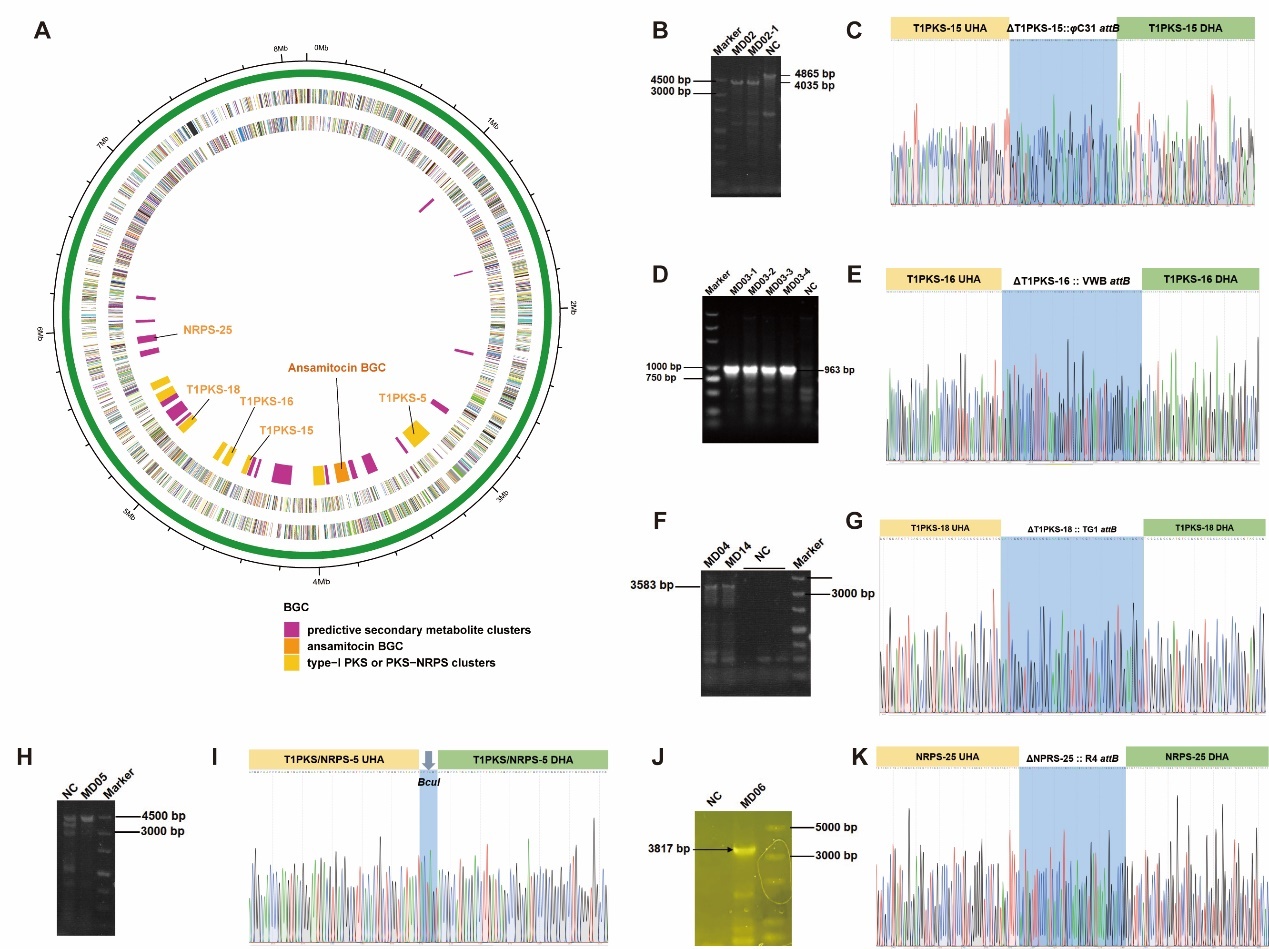


Fig. S5 Location of T1PKS gene clusters and identification of gene cluster deletion mutants

(A) Diagram of T1PKS gene cluster distribution was confirmed in parental strain L40. (B, D, F, H, J) PCR identification of mutant MD02, MD03, MD04, MD05, and MD06. (C, E, G, K, I) Sanger sequencing chromatograms for gene cluster deletion mutants. Up- and downstream homology arms are shown in yellow or green. Artificially inserted *attB* sites are shown in blue. NC, negative control. The mutant MD02 was deficient in T1PKS-15 with the *Φ*C31 *attB* site insertion. MD03 was deficient in T1PKS-16 and has the VWB *attB* site knocked in. MD04 has a knockout of approximately 50 kb fragment of T1PKS-18 with the TG1 *attB* site sequence insertion. MD05 has a knockout of T1PKS/NRPS-5 nearly 61 kb, introducing *Bcu*I, simultaneously. The 53 kb NRPS-25 fragment of MD06 was knocked out by insertion of the R4 *attB* site sequence.


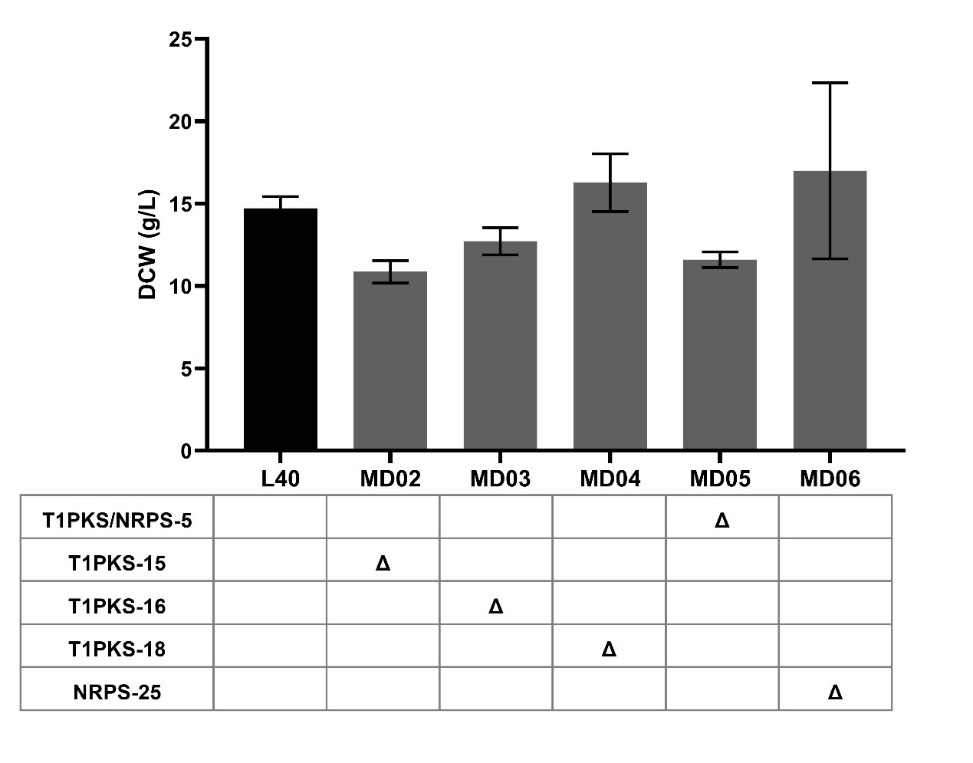


Fig. S6 Dry cell weight of gene cluster deletion mutants at the end of fermentation

L40, the parent strain. MD02, mutant with T1PKS-15 deletion. MD03, mutant with T1PKS-16 deletion. MD04, mutant with T1PKS-18 deletion. MD05, mutant with T1PKS/NRPS-5 deletion. MD06, mutant with NRPS-25 deletion. No apparent difference in dry cell weights (DCW) was noticed.


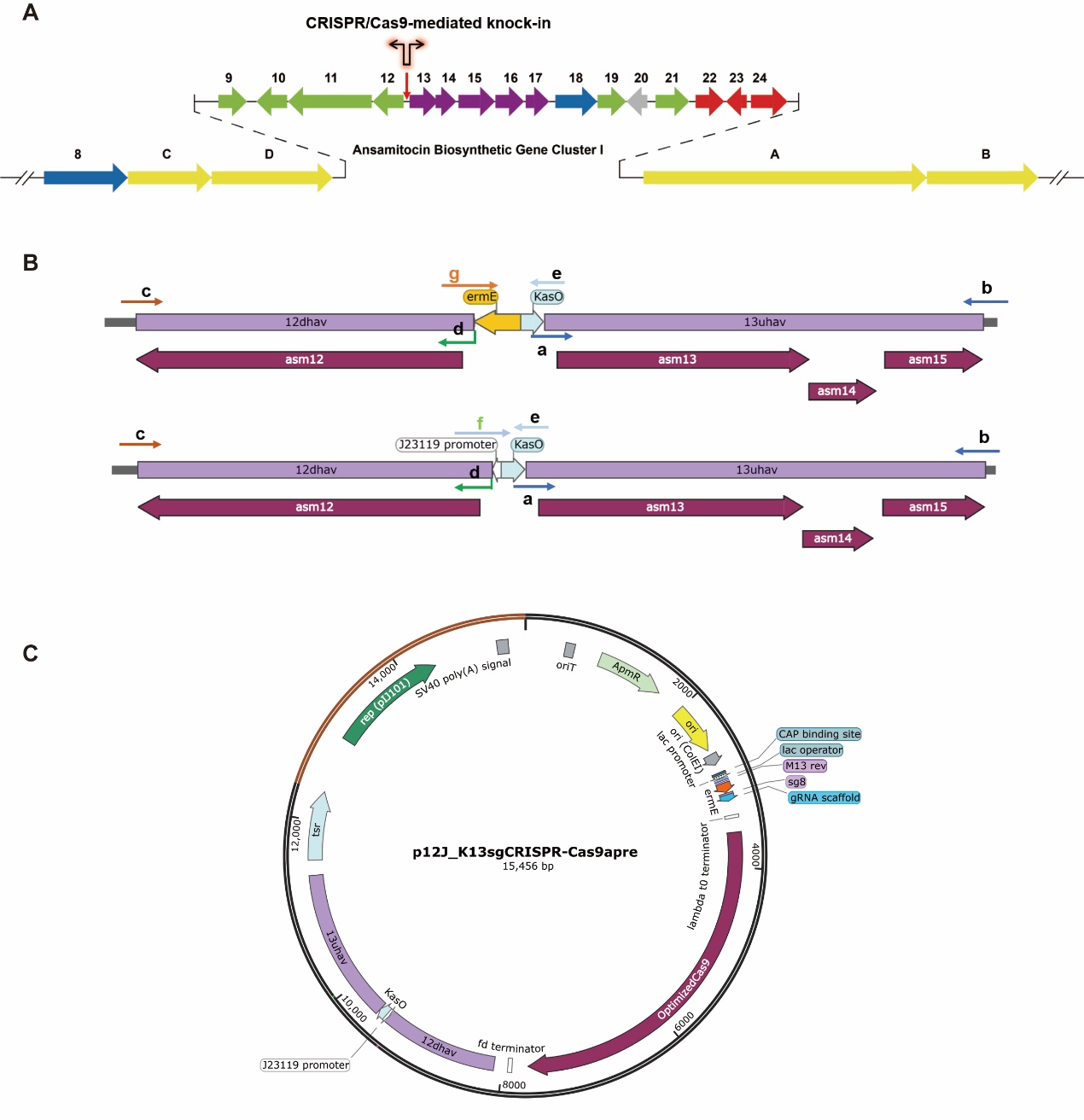


Fig. S7 Construction of bidirectional promoter knock-in mutant strains

(A) Schematic diagram of bidirectional promoter knock-in strategy. Bidirectional promoter was introduced into the *asm12*-*asm13* spacer, replacing the native promoter. (B) Assembly protocol for promoter DNA fragments and homologous arms. (C) Schematic overview of p12J_K13sgCRISPR-Cas9apre.


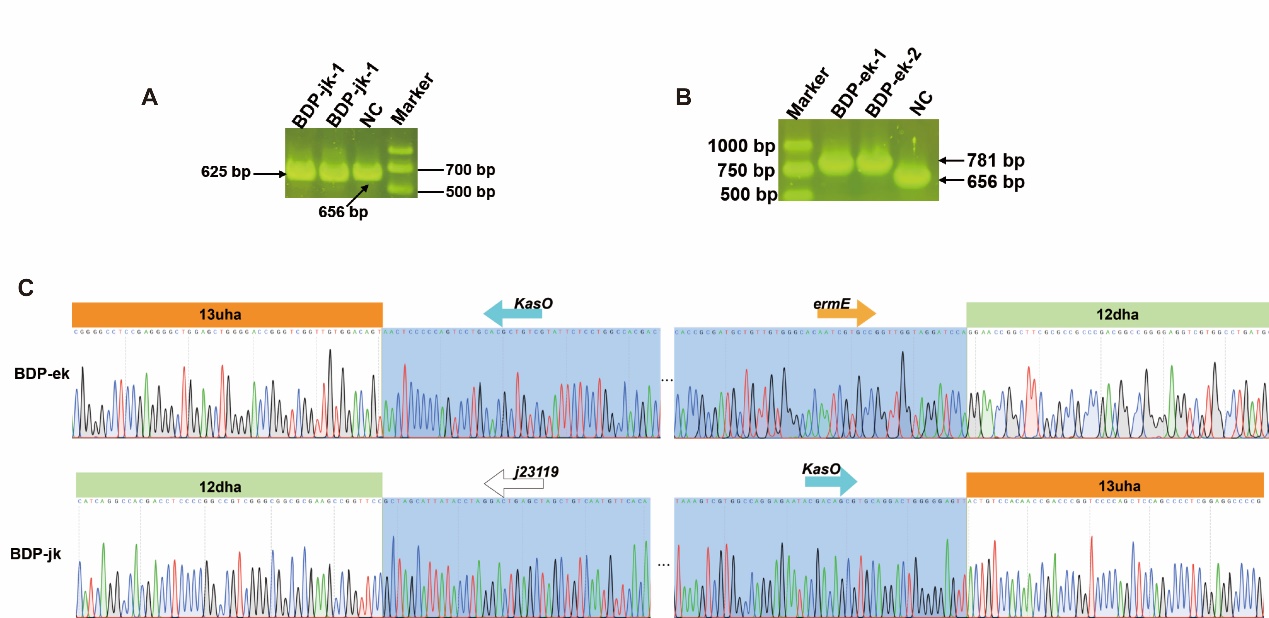


Fig. S8 Validation of bidirectional promoter knock-in mutant strains

PCR identification of bidirectional promoter knock-in mutant strain BDP-ek (A) and BDP-jk (B). NC, negative control. (C) Sequencing result of bidirectional promoter knock-in mutant strains.


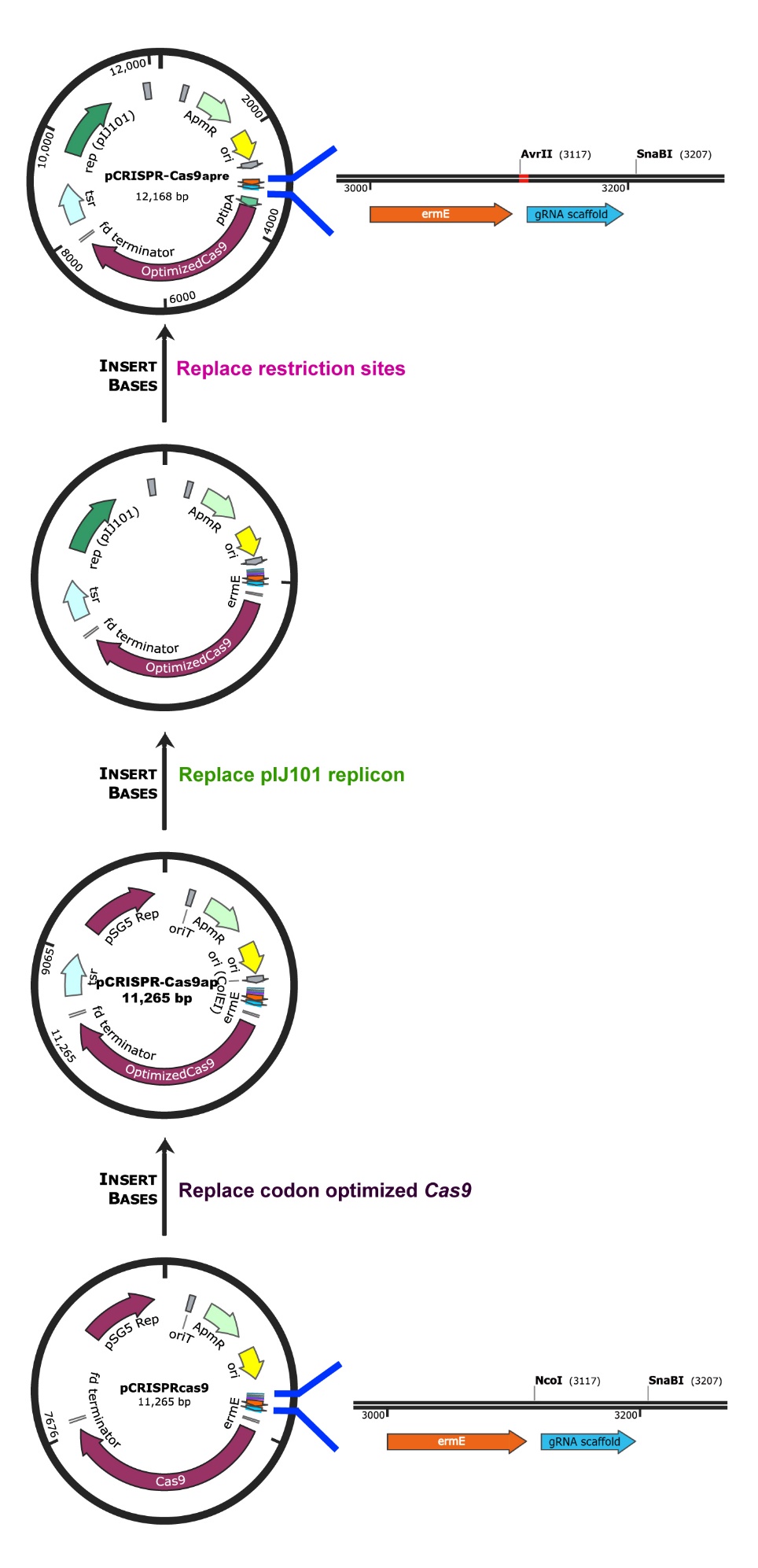


Fig. S9 Construction of pCRISPR-Cas9apre


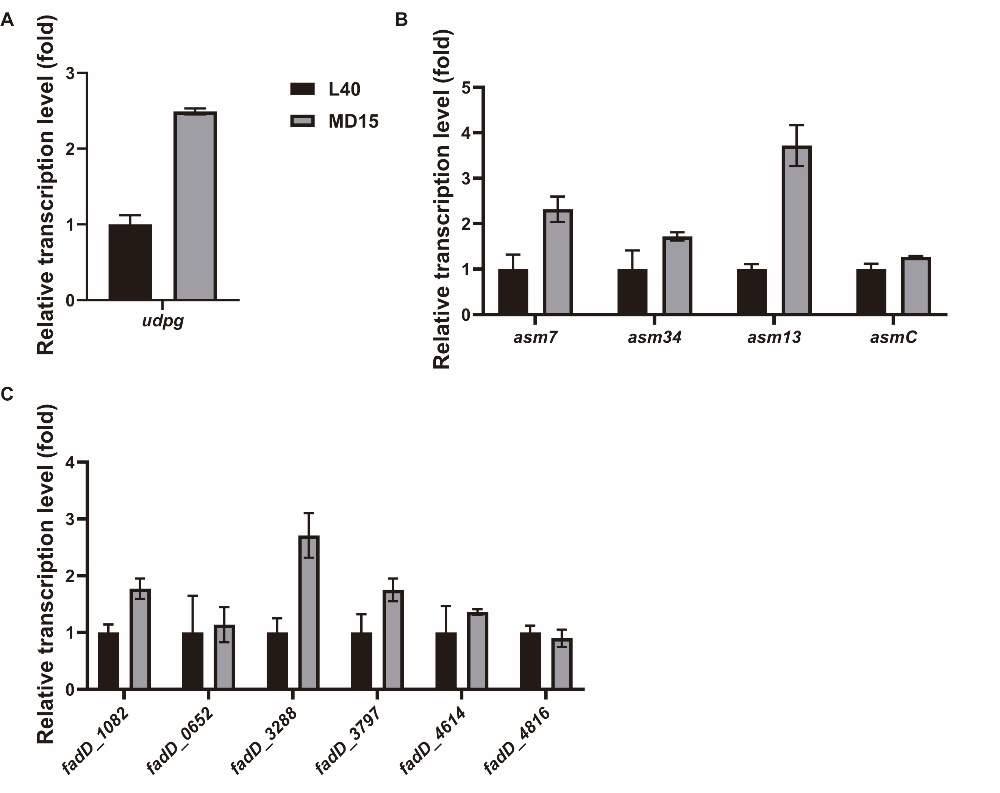


Fig. S10 Transcriptional analysis of *udpg* (A), AP-3 biosynthetic genes (B) and long-chain acyl-CoA synthetase genes (C) of strain MD15 at day 3 of fermentation

The *16s rRNA* gene was used as housekeeping gene, Fold change was normalized against the mean value of strain L40. Mean values of three independent experiments with SD (standard deviation) are indicated by *error bars*.


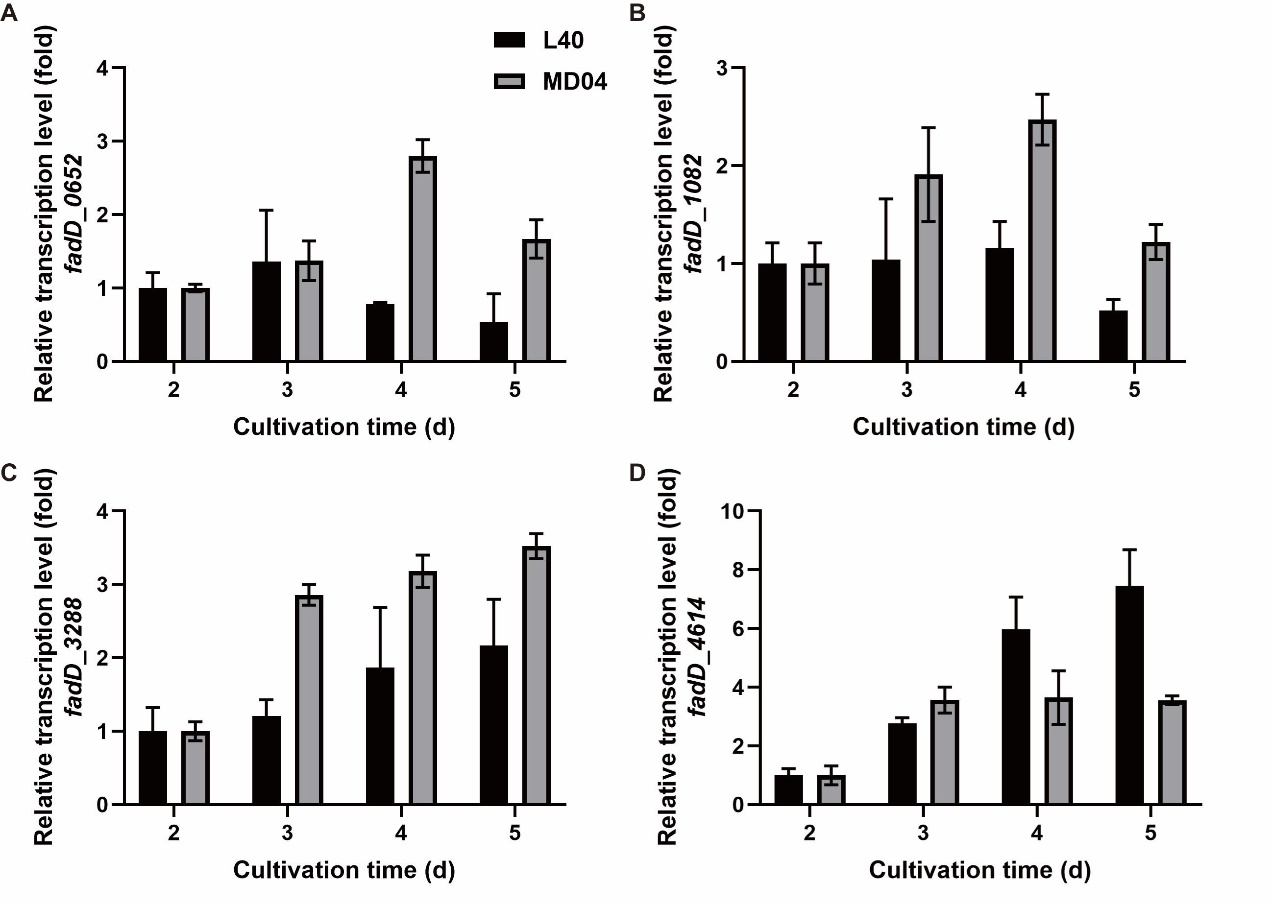


Fig. S11 Transcriptional profiles of fatty acyl-CoA synthetase genes in L40 (dark) and MD04 (gray)

Fold change was normalized against the mean value of day 2. Mean values of three independent experiments with SD (standard deviation) are indicated by *error bars*.

**References**

Li J, Guo S, Hua Q, Hu F (2021) Improved AP-3 production through combined ARTP mutagenesis, fermentation optimization, and subsequent genome shuffling. Biotechnol Lett 43:1143–1154. https://doi.org/10.1007/s10529-020-03034-5

Ning X, Wang X, Wu Y, Kang Q, Bai L (2017) Identification and engineering of post-pks modification bottlenecks for ansamitocin P-3 titer improvement in *Actinosynnema pretiosum* subsp*. pretiosum* ATCC 31280. Biotechnol J 12:1700484. https://doi.org/10.1002/biot.201700484

Paget Mark S. B., Chamberlin Leony, Atrih Abdelmadjid, Foster Simon J., Buttner Mark J. (1999) Evidence that the extracytoplasmic function sigma factor ςE Is required for normal cell wall structure in *Streptomyces coelicolor* A3(2). J Bacteriol 181:204–211. https://doi.org/10.1128/JB.181.1.204-211.1999

Sun Y, He X, Liang J, Zhou X, Deng Z (2009) Analysis of functions in plasmid pHZ1358 influencing its genetic and structural stability in *Streptomyces lividans* 1326. Appl Microbiol Biotechnol 82:303–310. https://doi.org/10.1007/s00253-008-1793-7

Tong Y, Charusanti P, Zhang L, Weber T, Lee SY (2015) CRISPR-Cas9 based engineering of actinomycetal genomes. ACS Synth Biol 4:1020–1029. https://doi.org/10.1021/acssynbio.5b00038
